# Mental health workers perceptions of disaster response in China

**DOI:** 10.1186/s12889-018-6313-9

**Published:** 2019-01-03

**Authors:** Yingjun Xi, Runsen Chen, Amy L. Gillespie, Yuyang He, Chihua Jia, Kuo Shi, Yiming Yao, Xin Ma, Wei Liu, Emily Ying Yang Chan

**Affiliations:** 1 The National Clinical Research Center for Mental Disorders & Beijing key Laboratory of Mental Disorders, Beijing Anding Hospital, Capital Medical University, No. 5 Ankang Lane, Dewai Avenue, Xicheng District, Beijing, 100088 China; 2 Department of Psychiatry, University of Oxford, Oxford, UK; 3 Collaborating Centre for Oxford University and CUHK for Disaster and Medical Humanitarian Response (CCOUC), The Jockey Club School of Public Health and Primary Care, The Chinese University of Hong Kong, Hong Kong, China; 4 Nuffield Department of Medicine, University of Oxford, Oxford, OX3 7BN UK; 5 Francois-Xavier Bagnound Center for Health and Human Rights, Harvard University, Boston, MA 02138 USA; 6 Advanced Innovation Center for Human Brain Protection, Capital Medical University, Beijing, China; 7 Department of Psychology of China Rehabilitation Research Center, Beijing, China

**Keywords:** Disaster response, Crisis intervention, Public metal health, Qualitative

## Abstract

**Background:**

The post-disaster mental health crisis intervention (MHCI) system in China remains immature and unsystematic. We aim to report the perceptions of a large sample of MHCI workers and government administrators and provide recommendations for developing a national mental health disaster response management plan in China.

**Methods:**

An in-depth qualitative study was conducted, collecting data from 20 focus-group discussions and 25 key stakeholder interviews. These recruited participants who had been involved in different types of disaster rescue across 7 provinces/cities where disasters have recently occurred. We used thematic analysis to analyze the data and relevant findings were extracted for policy recommendation.

**Results:**

Mental health workers’ perspectives were examined in detailed according to four core themes: forms of organization, intervention pathway, intervention strategy and technique, and public health information. Post-disaster MHCI should be approached in teams that are integrated with emergency medicine systems, and be led by unified command management. All levels of local health and family planning commission should prepare post-disaster MHCI work plans and build response teams/emergency centres. Future training for MHCI workers should focus on: building a sense of trust within the team; clarifying each member’s role; strengthening the screening, assessment and referrals training for psychological professionals; and providing psychological intervention training for Chinese psychiatrists. It is necessary to set up guiding principles for disaster research ethics, mental health rehabilitation and media interaction.

**Conclusions:**

Through exploring and analyzing the perceptions of current disaster response mental health workers and government administrators, our findings provide essential recommendations for developing a national to county level post-disaster MHCI emergency management plan and can guide the formulation of relevant laws and regulation in China.

**Electronic supplementary material:**

The online version of this article (10.1186/s12889-018-6313-9) contains supplementary material, which is available to authorized users.

## Background

In recent years, natural disasters, human-made disasters, terrorist attacks, and public health accidents have occurred frequently in China. The Chinese government faces new challenges in emergency management. Due to the important role of mental health crisis intervention (MHCI) in emergency management [1], the government is paying increasing attention to developing a MHCI system to respond to disasters at a national to county level.

Many developed countries have begun to incorporate MHCI into post-disaster relief efforts, and have developed effective systems [2–5], however in China the post-disaster MHCI system is still at an early stage of development. The first formal MHCI in China was performed for the Xinjiang Karamay fire in 1994 [6] and MHCI was implemented in Beijing for the 2003 SARS outbreak [7]. On the other hand, major application of MHCI at the governmental level to address potential mental health needs was not adopted until the 2008 512 Wenchuan earthquake [8, 9]. A temporary simple work plan (“The guiding principles of emergency mental health crisis intervention”) was issued by the Ministry of Health during the emergency in 2008 [10] and this initial guide was subsequently used for other emergency/crisis response such as the Kunming terrorist attack in 2014 [11], Ludian earthquake in 2014 [12], Ya’an (Lushan) earthquake in 2013 [13], sinking of a river cruise ship (Dongfang zhixing) in 2015 [14], Beijing flood in 2012 [15], Xinjiang Kashgar attacks in 2015 [16] Tianjin explosion in 2015 [17], and Yumen Plague in 2015 [18]. Although the 2008 guide played an important role in the preliminary standardization of post-disaster MHCI, as it was developed primarily to address natural disasters like Wenchuan earthquake, the generalization for application in other disaster contexts and how it might align with the rapid development of the disaster response capacity in China remains uncertain.

### Aims of the study

To facilitate future evolution and development of approaches related to post-disaster MHCI, the national health and family planning commission (Ministry of Health) set up this research team to conduct focus groups and individual interviews, with diverse MHCI workers and government administrators across 7 provinces/cities which had recently experienced different type of disasters. This study aimed to obtain the participants’ perspectives of the current post-disaster MHCI system, in order to provide recommendations for developing a national MHCI emergency management plan in China.

## Methods

### Study design

Between March 2015 and May 2016, the research team recruited its study participants through purposive sampling. Participants who had participated in MHCI for at least one disaster were selected from seven Chinese cities/provinces which had recently experienced different types of disaster - natural, public health, human-made or public safety disasters (as described in Table 1). Mental health workers were selected from each provinces/cities’ department of health and emergency management, health and family planning commission, and local mental health center, as well as government administrators from the department of health and emergency management in order to understand their management experiences of the fieldwork MHCI (See Table 2).

**Table 1:** Summary of selected disasters (data source fromWikipedia) for interview

Disaster	Summary
Wenchuan Earthquake	The Ms. 8.0 Wenchuan earthquake of May 12, 2008- the strongest earthquake since the establishment of People’s Republic of China (PRC)- caused great life and financial losses. Tens of thousands of people lost their homes and families. According to the Ministry of Civil Affairs, as of August 25, 2008, there were 69,226 people killed, 374,643 injured, and 17,923 missing. Immediately after the disaster, the Chinese government took several positive measures, such as providing financial support from the central government and 19 local provinces for reconstruction of the destroyed areas.
Ludian earthquake	The 2014 Ludian earthquake struck Ludian County, Yunnan, China, with a moment magnitude of 6.1 on 3 August. The earthquake killed at least 617 people, injuring at least 2400 others. As of 5 August 2014, 112 people remain missing. Over 12,000 houses collapsed and 30,000 were damaged.
Ya’an earthquake	On April 20, 2013, 7.0-magnitude earthquake occurred the Lushan County in the city of Ya’an, Sichuan province of China. This earthquake resulted in 196 people dead, 24 missing and at least 11,826 injured.
Kunming station attack	In the evening of March 1, 2014, a knife attack occurred inside the Kunming Railway station in Kunming, Yunnan, China. At around 21:20, a group of 8 knife-wielding men and women attacked passengers at the city’s railway station. Both male and female attackers pulled out long-bladed knives and stabbed and slashed passengers. The incident, targeted against civilians, left 29 civilians and 4 perpetrators dead with more than 140 others injured.
Beijing flood	On Jul 21, 2012, Beijing experienced one of the heaviest rain events in the past 60 years. The heavy rainfall triggered flash flooding and landslides, which killed 79 people and caused US $2 billion in direct economic losses, destroying at least 8200 homes in the city and affecting more than 1.6 million people.
Kashgar attacks	On July 30 and 31, 2011, the Kashgar attacks were a series of knife and bomb attacks in Xinjiang province, China. This attack resulted in 23 people dead and at least 42 injured.
Sinking of Dongfang zhi Xing (Eastern Star)	Dongfang zhi xing was a river cruise ship that operated in Three gorges region of inland China. On June 1, 2015, the ship was traveling on the Yangtze River in Jianli, Hubei Province with 454 people on board when it capsized in a severe thunderstorm. On 13 June, 442 deaths were confirmed, with 12 rescued. It is the deadliest peacetime maritime disaster in China’s history.
Tianjin Explosion	A large explosion in Tianjin occurred on August 12, 2015 at approximately 23:30 and was followed by a chain of explosions that killed 173 people (eight missing) and injured more than 700. The location of the explosions was a container storage station at the port of Tianjin where there were over 40 types of hazardous chemicals being stored according to related reports. These chemical included potassium nitrate, sodium nitrate and sodium cyanide. The complex and very toxic chemicals (including cyanide materials) made these explosions much more complicated than any other common explosion with regard to the condition of the injured patients.
Yumen plague	Bubonic plague is a bacterial infection known as Black Death, a virulent epidemic that killed tens of millions of people in fourteenth century Europe. On July, 2014, tens of thousands of people were trapped in Yumen city, in the north-western province of Gansu when officials swiftly locked down the city after a man died of plague. 151 people were placed in quarantine. No further plague cases have been reported in Yumen.

**Table 2:** Summary of participants’ affilitation

Participants affiitations	Location
Office of Health Emergency, National Health and Family Planning Commission	Beijing
Beijing Anding Hospital, Capital Medical University	Beijing
Peking University Sixth Hospital (Institute of Mental Health)	Beijing
Capital Normal University	Beijing
Beijing Huilonggun Hospital	Beijing
Tianjin Mental Health Centre, Tianjin Anding Hospital	Tianjin
Health and Family Planning Commission of Tianjin	Tianjin
Kunming Medical University affiliated Yunnan psychiatry hospital	Kunming, Yunnan
Mental Health Center of Yunnan Province	Kunming, Yunnan
Yunnan Public Security Bureau	Kunming, Yunnan
Kunming Public Security Bureau	Kunming, Yunnan
Kunming Traffic Management Bureau	Kunming, Yunnan
Kunming Crisis Intervention and Research Center	Kunming, Yunnan
Yunnan Health Education Institute	Kunming, Yunnan
Yunnan Kunming Young mental health help hotline	Kunming, Yunnan
Yunnan Third People’s Hospital	Kunming, Yunnan
Yunnan Red Cross	Kunming, Yunnan
The Emergency Center of Yunnan Province	Kunming, Yunnan
Health and Family Planning Commission of Yunnan Province	Kunming, Yunnan
Ludian Center for Diseae Control and Prevention	Ludian, Yunnan
Yunnan Armed Police General Hospital	Kunming, Yunnan
Yunan Second People’s Hospital	Kunming, Yunnan
Renmin Hospital of Wuhan University	Wuhan, Hubei
Wuhan Mental Health Center	Wuhan, Hubei
Health and Family Planning Commission of Hubei Province	Wuhan, Hubei
Wuhan Public Security Bureau	Wuhan, Hubei
Jingzhou Mental Health Center	Jingzhou, Hubei
Huaxia Mental Health Education Center	Wuhan, Hubei
Wuhan Chuxing Social Work Service Center	Wuhan, Hubei
Center for Diseae Control and Prevention	Yumen, Gansu
Renmin Hospital of Yumen	Yumen, Gansu
Xihua University	Chengdu, Sichuan
University of Electronic Science and Technology of China	Chengdu, Sichuan
Chengdu University of Traditional Chinese Medicine	Chengdu, Sichuan
The First People’s Hospital of Kashgar	Kashgar, Xinjiang

Given the large number of mental health workers and time limitations, we predominantly chose the methodology of focus group interviews. A total of 20 focus groups, each with 4 to 16 participants, were conducted (Table 3 Participant characteristics); each focus group lasted for 90 min to 2 h in local mental health centers, general hospitals or government offices. However, in towns where the mental health service and local government were small, or in scenarios where participants had conflict schedules with our focus-group sessions, individual interviews were also conducted – this allowed some more in-depth exploration of these individuals’ experiences. In total, 25 individual interviews were conducted (Table 4 Participant characteristics); these lasted for 40 to 60 min. All participants were required to complete a demographic questionnaire at the beginning of each interview. The focus groups and individual interviews were conducted by Mental Health Crisis Intervention and Stress Management Center affiliated Beijing Anding Hospital staff. All staff had been trained in facilitation of focus groups and individual interviews. To ensure we had developed a comprehensive interview guideline to reflect the mental health workers’ experience and views, we conducted a systematic literature review. Using Pub-Med, Web of Science, PsycINFO, we searched for scientific literature, guidelines, and manuals in English or Chinese language using search terms including “disaster”, “emergency”, “mental health”, “public emergency”, “Mental Health Crisis”, “mental health Intervention”, “psychological first aid”, “expert consensus”, and “guidelines”. The interview guideline was also reviewed by the local experts. Please see Additional file 1 for the complete interview guideline.

**Table 3:** Summary of participant (focus group) characteristics

	Total (*n* = 166)
Demographics	
Age (range, years)	25–57
Male	87 (52.4%)
Female	79 (47.6%)
Education	
Junior College	29 (17.5%)
Undergraduate degree	88 (53%)
Master degree	34 (19.9%)
PhD degree	17 (9.6%)
Career background	
Psychological counsellor	56 (33.7%)
Psychotherapist	14 (8.4%)
Psychiatrist	50 (30.1%)
Social worker	12 (7.2%)
Nurse	16 (9.6%)
Government administrator	18 (10.8%)
Numbers of participation in selected disaster rescue	
Once	76 (45.8%)
Twice	72 (43.4%)
More than Twice	18 (10.8%)
Focus group location (number of groups)	20
Beijing	4
Tianjin	1
Kunming, Yunan	6
Ludian, Yunan	2
Wuhan, Hubei	3
Chengdu, Sichuan	2
Yumen,Gansu	2

**Table 4:** Summary of participant (individual interview) characteristics

	Total (*n* = 25)
Demographics	
Age (range, years)	33–58
Male	17 (68%)
Female	8 (32%)
Education	
Junior College	3 (12%)
Undergraduate degree	10 (40%)
Master degree	8 (32%)
PhD degree	4 (16%)
Career background	
Psychological counsellor	3 (12%)
Psychotherapist	2 (8%)
Psychiatrist	6 (24%)
Government administrator	14 (56%)
Numbers of participation in selected disaster rescue	
Once	8 (32%)
Twice	12 (48%)
More than Twice	5 (20%)

### Data analysis

All interviews were audio recorded and data transcribed verbatim by a professional transcription company. All Chinese transcriptions were translated to English by a professional translator. In addition, one researcher (AG) corrected grammatical errors. Data were analyzed by manual thematic analysis based on the research questions, in order to identify the themes. There are six phases to thematic analysis technique: familiarizing oneself with the data; generating the initial codes; searching for themes within text; reviewing the themes; defining and naming the themes; and producing the report. Two researchers (XY and CR) independently reviewed the coding of the themes, and held several meetings to compare and discuss their notes in order to achieve a consensus upon the themes and an accurate interpretation of the data [19]. Dual researchers reviewed the transcription of the taped collected data.

### Ethics

The study protocol and consent form were approved in written from the Human Research and Ethics Committee of Beijing Anding Hospital, Capital Medical University. All participants were informed of the objective of the study and gave written consent before the investigation.

## Results

**Fig. 1:**
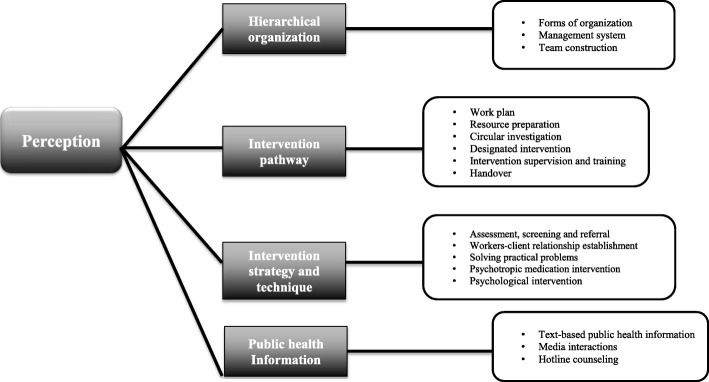
Summary of theme and sub-theme

For further examples and details relating to each theme please see Additional file 2. For a summary of theme and sub-theme please see Fig. 1.

### Hierarchical organization

#### Forms of organization

Mental health workers suggested that MHCI should be integrated with overall rescue and emergency medicine response systems instead of conducting their work independently. Additionally, MHCI teams should stay up-to-date with information from the primary rescue response teams, so that they can perform effective and scientific MHCI fieldwork.

*“The problem was that our mental health intervention work did not follow up with the overall rescue work. We were not under the same working arrangement, lacking clear information”.*

Many participants mentioned that the local mental health interview team should lead the intervention as the primary force, with support from the external team.

*“It would be too late if the external intervention team takes a long time to come here”.*

#### Management system

Overall, respondents regarded major improvement had been observed in post disaster MHCI. One improvement has been the shift from an approach of “individual combat” to the promotion of MHCI team cooperation. Effective team cooperation depends upon a unified and powerful management organization, an effective team provides stability for MHCI workers, and this stability consequently provides benefits for the victims being supported.

*“In my view, the MHCI work for the Wenchuan Earthquake was a complete mess. Everyone was doing their individual job without a holistic work plan or scheme, and no unified management.”*

*“I think the psychological intervention after an accident is different from my normal routine job. Once arriving at the site, I was suddenly dumb, knowing nothing about what I should do. My immediate reaction was to find the organization and accept my task through organization. I felt much more at ease when the rescue command center assigned me to work with the mental health intervention team organized by local medical institution. I could finally carry out my work without worries. Therefore, an organization is of vital importance; it settled me down at the beginning.”*

#### Team construction

##### Team leaders

The effectiveness of the MHCI depends largely on the abilities of the team leader. This is embodied in the quality of their management, organizational skills, and professionally. Additionally, good MHCI leaders were associated with particular personality traits, especially modesty and trustworthiness.

*“No one knows how this team would work if there was not an excellent leader whom everyone respected. It is possible that everyone would ignore each other and everything would end with a mess.”*

*“This leader deserves its name. He is indeed our role model. Not only for being amazing at work, he also shows perfect personality, with a great approach for dealing with people. Everything goes on well as long as he is present, and we understand and support each other during group discussions. In fact, to some extent, whether our work can meet the expectations depends on the leader’s encouragement and model effect.”*

##### Team experts

As reported by the respondents, external team experts mostly came from the national crisis intervention team who have abundant experience. These experts provided guidance and assistance to the local MHCI team. Respondents commented that the establishment and operation of expert groups had and could facilitate the overall MHCI work. Many MHCI workers agreed that this primarily depends on creating a group with a sensible composition of experts and the personal qualities of the experts.

*“What left me with a strong impression is the way that the mental health intervention expert group worked out. In that mental health intervention mission, the psychologists appointed by National Health and Family Planning Commission suggested that local hospitals and university volunteer organization mental health intervention team select several cooperative and professional experts to form an expert group. In that mission, this group played a fundamental role. It gathered the problems we met in work, then offered advice to these problems one by one, and finally adopted the consensus decisions. This also reflected the centralized democratic manner of working.”*

*“After the accident, we soon established a psychological intervention team. Although we did not have much experience, external rescue experts have offered much support and encouragement, helping us to make work plans and other mental health intervention materials, and taught us how to do the intervention from the beginning. This laid a solid foundation for later psychological rehabilitation. Therefore, our work became increasingly familiar and proficient.”*

##### Team members

MHCI workers discussed the abilities and competencies required for their role. Physical, mental and mutual trust among team members were reported to be key determinants. Some MHCI workers mentioned that collaborating with team members from different disciplines caused tension and affected their enthusiasm for collaboration. Some workers noted their personal limitations and the need to improve their coping mechanisms for crisis intervention fieldwork.

*“We felt that it was very hard to cooperate with psychiatrists. They seemed to look down upon us and I think they only know how to prescribe medications.”*

*“I could still remember that when I first joined this work in Jingzhou, it felt uneasy and worrying. I had no previous experience. MHCI fieldwork is completely different from my everyday work. I would have liked to have a deeper background and more abilities in psychology.”*

*“Our local Mental health workers lack adequate abilities to cope with crisis intervention for the clients. They urgently need further training from an experienced expert.”*

#### Intervention pathway

Based on the descriptions of MHCI workers, this paper presents a six stages MHCI intervention pathway from work plan through to handover.

#### Work plan

MHCI workers mentioned the importance of formulating a work plan and corresponding documentation. When faced with a new working environment, only having access to vague information about the role increases anxiety. Therefore, formulating a clear work plan with guidance and requirements – including working principles and clear job duties of each MHCI member – provides reassurance and stability. However, many local governments do not yet have emergency work plans for post-disaster MHCI.

*“Being the leader of the intervention team, I felt under more pressure. My responsibility was to establish a bridge between the crisis intervention decision-makers and the MHCI worker. I needed to make a plan for myself, as well as for other team members, so that everyone could work under regulation without any omission, and we could save time avoiding repetitive notifications. This also made the working procedure more standard.”*

*“We need to follow relative requirements and principles in every day psychological counselling and psychotherapies. However, MHCI for public accidents, apparently, calls for special requirements, and we need to be clear about this. Because I understand that the success of MHCI does not only rely on psychological factors, additional factors such as social, humanistic, and even medical factors, also play a role. Therefore, the formulation of relative documents must be comprehensive. Any missing parts might cause the intervention to become chaos.”*

#### Resource preparation

Resource preparation consists of providing the necessary materials to support two key aspects: MHCI workers’ day-to-day living arrangements, and working equipment. MHCI workers referred to experiences of chaos caused by insufficient resource preparation and therefore stressed the importance of this important aspect of work.

*“Our hospital always puts MHCI at priority level, being prepared for material supply in peacetime, and doing regular checks and updates. Because of this, once an accident occurs, we are soon able to grab the materials and arrive at the site. Actually, the key to this work lies in daily preparation. Without careful daily treatment, it is impossible to be responsive to emergencies. This substantiates the professionality of the MHCI team of our hospital.”*

*“As the sky turned dark, we still were unable to offer any help and everyone became hungry. We then had to settle down at a temporary resettlement point, and received some food together with other victims. Even now I feel embarrassed about it.”*

#### Circular investigation

Circular investigation refers to circulation of the disaster site and nearby locals for information-gathering and provision of initial practical support. MHCI workers universally recognize this aspect of interventions from their past practical experiences, and comment that continued circular investigation throughout the intervention can bring a sense of stability to disaster victims.

*“The circular intervention in my definition is visiting the victims with a helpful attitude as a professional, detecting and solving the problems.”*

*“Once when I knocked on the door of this family and introduced ourselves to them, they treated us with bad attitudes, saying ‘get out, get out, we don’t need your help.’ We felt very uncomfortable, but our leader told us that this was the point of our work. While observing the way people communicate with us, we could learn that how this accident influenced this family. Therefore, we must show our tolerance and understanding.”*

#### Designated intervention

Designated intervention refers to the work of MHCI workers in establishing a steady relationship with clients and offering long-term professional mental health treatment. MHCI workers mentioned two forms of designated intervention. The first is the mental health services provided by psychiatric professionals at mental health institutions, the second is the mental health services provided by psychological professionals at a client’s home.

*“Circular investigation and designated intervention together contribute to a satisfactory mental health intervention. We arrive at a location, and visit the families one by one. Whenever we spot any emotionally unsteady clients, we find out more about him through observation or chatting with his family, and report to the leader.”*

*“In one investigation task, we found someone with abnormal behaviors at the resettlement point, with salient depressed mood and glazed eyes. We then entered his house and found a rope. We immediately got in contact with his family and learned that he once suffered from bipolar disorder. According to the psychiatric assessment then conducted, there was major depression going on, and we thought he was of high suicidal risk. After patient communication with his family, he was referred to a local mental health center and taken care of.”*

#### Intervention supervision and training

To ensure the quality of MHCI, effective training and supervision of MHCI workers is required as the rescue/relief operation evolves. Specifically, a regular meeting system, a training scheme, and a supervision schedule are necessary as precautions for preventing, identifying and solving problems as early as possible in the fieldwork.

*“Actually, initially, I didn’t care much for regular meetings. After a whole day’s work, all I wanted to do is to have a rest. Also I thought there is nothing wrong with my duties, so no need to report again. It’s a waste of time. However, the meetings were required by the expert group, so I had to attend. After attending, I realized that it is worth doing. First, I found out that there were some minor deficiencies I can improve, thanks to observations from other colleagues. Second, everyone possesses some values which I could learn from, and it was an enjoyable experience to share my opinions with the team. In sum, the meeting process is a place to share knowledge and offer mutual encouragement.”*

*“I received short training in mental health crisis intervention at the beginning of work. This indeed was helpful, as it clarified many of my questions and relieved my anxiety. After all, MHCI differs in many ways from my daily job. In the meantime, I wish the training could involve more case analysis and role playing. Maybe this would help me to better understand the principles. In addition, I think training should be divided into stages. At the initial stage, just telling us what is correct and what is wrong. Later we can include deeper discussion and training according to the developmental pattern of crisis client’s mental state. At the final stage the training could tell us about sadness and trauma treatment.”*

#### Handover

Handover is the process during which one staff explains his or her duties and other work-related situations to another staff before his or her responsibility terminates (e.g., after a two-weeks’ working contract), so as to assure uninterrupted crisis intervention services. MHCI workers showed concerns specifically about the last stage of MHCI; due to various factors, this is frequently handled poorly. They suggested that it is necessary to develop a plan for tracking follow-up of victims and disaster mental health rehabilitation in China.

*“As the overall rescue work has come to an end, however, there is no subsequent psychological rehabilitation scheme for these clients. Although we try to do something; there seems to be no alternative ways.”*

*“I think the follow-up work of MHCI is as important as the emergent stage. Yet due to many internal and external factors, this duty has not received enough attention, therefore its implementation is not ideal. In fact, the follow-up of MHCI involves various types of work, for example the trauma therapy for crisis clients, scientific research on group disaster community rehabilitation, and policy research on future MHCI management, etc.”*

#### Intervention strategy and technique

##### Assessment, screening and referral

Assessment, screening, and referral are the core procedures mental health workers need to follow. They first conduct a thorough assessment on client’s psychological state (assessment), sort them into groups according to their symptoms (screening), and come up with different intervention strategies (referral). MHCI workers mentioned issues regarding the screening and assessment of clients. Many lacked a clear and correct understanding of these tasks.

*“We met with serious trouble once during the MHCI work after the shipwreck. One lady’s husband had died in this accident. Upon hearing this news, the lady presented with serious psychotic responses, claiming that her husband was waving his hand towards her on a small island far away, and she could hear his shouting at her. This was typical illusion and delusion. After careful consideration, we made the decision to refer this lady to a mental health institute for further psychiatric diagnosis and treatment.”*

*“I used to treat assessment and screening as the same procedure, but now I believe that these two should be separated.”*

Some MHCI workers stated that questionnaire assessment and interventional research should be based on clients’ interest and permission, and that a process of research ethics must be created and followed.

*“Although taking questionnaires showed their prudence in work, a lack of humanistic care should still be considered unethical.”*

*“We could never force them to participate in the research, which should be based on crisis clients’ permission.”*

#### Worker-client relationship establishment

MHCI workers discussed their strategies for establishing and maintaining cooperative relationships with crisis clients, noting that the goals required for different stages of the disaster response vary.

*“I think in MHCI, the most significant part is how to establish a good relationship with the clients at the beginning.”*

*“When first approaching the clients, the first priority is to win their trust.”*

*“To tell the truth, most clients I engage with gradually recover to a rational and peaceful state. As our mutual understanding moves forward, they change from being initial passive and helpless to initiating communication with us, and starting to plan for the future. Now, being MHCI workers, not only should we continue our support, but also show full respect to these clients. We need to think twice about our positions and realize that we are just a supportive role, so that we can further improve crisis client’s initiative.”*

#### Solving practical problems

MHCI workers frequently talked about their experiences with solving practical problems for crisis clients. They agreed that problem solving should follow the principles and professional standard of clinical psychology practices, using strategies and techniques based in psychological understanding.

*“Social work is my major. I remember that I was very confused during my first experience of MHCI. I admired my psychiatrist and psychologist colleagues, who could make use of their expertise, while my responsibilities seemed not important at all. The leader of our team seemed to realize my depression, saying ‘Every single MHCI worker plays his or her role. As for a worker with a social work background, you should substantially fulfill your duties, which are helping victims to solve their practical problems. This requires professional knowledge, and is never easy-peasy.”*

*“As a psychiatrist, we share some advantage in practical MHCI work. Because, you know, the most important responsibility for clinical work is to guarantee patient’s life safety. Therefore, in MHCI, we are always cautious about this, observing closely the mental conditions of the patients to see if they are related to their injuries. Once potential risk has been spotted, we will handle it immediately. I think this is an important part of crisis intervention.”*

#### Psychotropic medication intervention

Most of the interviewed MHCI psychiatrists mentioned psychotropic medication intervention, including discussion of the ideal availability and usage of psychotropic medication, types of medication, and importance of prescription notes.

*“Although the majority hold that crisis client’s mood swings do not require medication, I think this does not apply to all situations. For example, three days after the accident, some people still cannot have a good night sleep, even though they have been settled at a safe place. Therefore, medication treatment is necessary, but must be assessed by a psychiatrist.”*

*“The medication treatment in MHCI clearly differs from psychiatric clinical treatment. Although the dose of the medication in the overall working period is not large, if there is a mistake with the medication, serious consequences might occur. Therefore, I suppose that we must take care of every single detail of medication treatment. For example, specifically recording the medication and personnel, make sure everything is safe.”*

#### Psychological intervention

During intervention, crisis intervention workers need to pay attention to clients’ emotions, cognition, and behaviours, and offer appropriate treatments accordingly. Major intervention techniques include Mood Stabilizing, Cognitive Integration, and Behavioral Leading. Mood Stabilizing requires workers to acutely identify and understand clients’ emotions, provide sympathetic responses, and lead them to a calm and rational state at the right moments. Cognitive Integration is a technique in which crisis intervention workers help the clients to understand the abnormal symptoms they are experiencing and the whole picture of the event. This includes pointing out how their negative thoughts affect their behaviors and distort their cognition; strengthening their positive and optimistic thoughts, etc. Behavioral Leading is a more active and suggestive technique, in which workers perform activities such as muscle relaxation, positive implantation, trauma isolation, and crisis desensitization. However, these methods require professional training in psychological treatment.

*“A stable emotional state is the most important thing for crisis clients after public accident. However, this goal is not easy to achieve at the beginning, and neither is it a practical goal. The victims I witnessed were usually either crying, or terrified, or completely silent, or showing impulsive behaviors. We must stay calm in these messy situations. Not try to persuade them to do anything, instead we should accompany them, staying by their sides. Using their body reaction to understand their feelings, and using practical actions to protect, support them. Because when people are involved in the emotional waves caused by natural disasters, our work at a cognitive level, e.g. persuasion, explanation, will be futile. It is malposed communication.”*

*“I notice that crisis clients are often not able to talk about the complete picture of their accident experiences. They tend to exaggerate the horrible scenes they witnessed and their helplessness under that condition. In fact, many positive elements in the accident are missing. These positive elements include not only crisis client’s calmness and persistence during crisis, but also the support and help they received from others. Therefore, during intervention, what I need to do is to restore this content at a cognitive level, diluting their negative thoughts.”*

*“Relaxation techniques are frequently adopted in MHCI. Because MHCI workers will often need to deal with crisis clients who present with tense emotions or sleeping disorders after accidents. However, many workers are rather rigid and stiff when performing this technique, ignoring the principles and procedures. This has resulted in poor efficiency of the technique and even some side problems. Therefore, the implementation must be based on crisis client’s absolute trust in MHCI workers, and the worker should be wary about the opportunity for implementation, following the principles and correct procedures.”*

#### Public health information

##### Text-based public health information

MHCI workers said that they commonly prepared mental health material for public dissemination before departure, as well as disseminating mental health brochures to the disaster victims during circular investigation and putting up posters in salient places.

*“Four cornerstones of health education are healthy diet, regular exercise, limiting cigarettes and alcohol, and mental harmony. These tips should be remembered, because after the crisis, many victims lose their regular life and eating habit. The loss of finance or of family members drives them into deep agony and uneasy. Some might drown their worries in drink, and be in a mentally uncontrolled and unbalanced state. Therefore, every one of our MHCI team should hold a general idea about health, offering health education properly, since this is the premise to help crisis clients to recover to a normal state soon.”*

#### Media interactions

MHCI workers frequently commented on media response to disasters; some argued that some media practices may impede the implementation of MHCI and be harmful to victims’ mental health.

*“I have an aversion to the media. This indeed interferes with our work and efficiency.”*

*“Sometimes the journalists follow us everywhere we go to. Although I completely understand this is their job, they really know nothing about the specialty of our MHCI work. The priority of our work is not to interrupt the crisis clients, yet these journalists do not follow this approach, always directly asking them about sensitive issues. Some residents are not willing to answer [media requests], and [media] still keep on asking. In fact this is of harm to the victims.”*

#### Hotline counseling

Another method for disseminating mental health knowledge to the public is hosting a psychological hotline. MHCI workers believed that a hotline could not solve complex problems, but could offer scientific explanation and guidance on some psychological phenomena and that hotline counselling is a valuable way to improve MHCI services and information access.

*“Hotlines are good. They not only help to handle crisis situations, but also strengthen later periods of psychological rehabilitation work. However, this job requires people, material, and money. If these cannot be guaranteed, this job is hard to continue for long. Therefore, this cannot be decided by MHCI workers.”*

## Discussion

Our qualitative study findings are consistent with current knowledge of MHCI development which recommended that the psychosocial care after disasters should be integrated within existing procedures for crisis management [20]. Our findings support the proposition that MHCI teams should be integrated with emergency medicine systems. After a disaster, the community near the disaster site is likely to be severely damaged. Whether MHCI teams can successfully provide an efficient post-disaster deployment of mental health interventions relies heavily on a comprehensive and accurate understanding of the ongoing condition and development of the disaster, enabling team discussion of the overall psychological intervention strategy and the appropriate plans and programs. For example, required information includes: the type of disaster (natural disaster event, man-made disaster or public health accident); geographical range of the disaster; population affected; local cultural characteristics; the centralized location of affected people; basic facilities and resources for a local mental health intervention; information about other mental health colleagues; and the specific difficulties related to this mental health intervention.

Furthermore, a recent systematic review of mental health responses to community disasters suggested that mental health intervention should be integrated into emergency medication and trauma care response [21]. Usually when a disaster occurs, mental health workers from non-governmental organizations, volunteer organizations, private psychological organizations, universities, and hospitals will arrive at the event site and volunteer to join the rescue work [22]. Those who are equipped with some psychological or sociological background might be subsequently assigned to mental health intervention, however, many volunteers lack experience working in crisis mental health intervention and some lack any systematic training in psychology. In addition, mental health workers are unsystematically located across the event site resulting in a chaotic situation. The lack of coordination means some disaster victims receive multiple mental health counseling from different crisis intervention workers of varying skill level, which can be damaging and compound existing trauma. This is another significant reason that building a managing organization, under the same command center as the emergency health department and bound with the emergency medicine system, is a key priority for mental health interventions.

MHCI should be conducted by multi-disciplinary teams, because different workers have their own limitations, often self-identified. For example, there is insufficient psychological training for psychiatrists in China (with no systematic training in clinical psychology for medical trainees majoring in psychiatry) but psychiatrists are normally the mental health workers recruited by medical institutions. When psychiatrists are dispatched to crisis sites, they need to care for clients who are distinct from those they meet in their routine hospital work. In contrast, workers with psychological backgrounds often lack the training and knowledge about screening, assessment and referral which is familiar to psychiatrists, as they receive no psychiatric training and do not work in psychiatric hospitals. Therefore, when dealing with clients experiencing an emergent mental disorder that requires medical treatment, they may not have the experience necessary to evaluate the situation. If they can correctly diagnose a client, they may still be unfamiliar with the subsequent processes such as referral and intervention. Therefore, working in multi-disciplinary teams can help ensure successful progression of mental health interventions post-disaster and protect the physical and mental health of every team member. Collaborative approaches to MHCI are necessary and mental health workers should consistently trust and encourage each other, regardless of background and training. This result suggest that future training courses should concentrate on a) building trust within MHCI teams, b) teaching strategies for screening, assessment and referral to workers with psychological backgrounds, and c) providing education in psychological intervention techniques for Chinese psychiatrists.

A MHCI team normally includes nurses, psychiatrists, social workers, psychotherapists, psychologists, and counselors, however this is less consistent in China. Typically, all team members provide the same psychological intervention to clients, with an expectation that psychiatrists play the unique role of medication prescription and experts provide supervision and guidance for the team. The European Network for Traumatic Stress (TENTS) guidelines have suggested that each member of a MHCI team should have an understanding of their role and relevant laws and regulations [3].

In Japan, many local government departments of mental health have a disaster response guideline [2], but this is not true for China. This study urgently suggests that all levels of the local Health and Family Planning Commission should prepare a local emergency post-disaster MHCI plan and relevant documentation, as well preparing necessary resources and equipment in order to enable a prompt emergency response. Immediately after a disaster, MHCI teams must promptly formulate appropriate documents, specific to the situation, providing guidance for team members to perform their duties. Documents should include working regulations and guiding principles, all necessary template report forms, psychological records, and team member records. Specially, regulations should cover privacy protection for the clients, legal requirements for MHCI workers, the establishment of an information report system, and a supervision and training scheme. The preparation should also establish working requirements of the team, working processes, and mental health intervention techniques. These documents should be formulated within 24 h after the disaster by a core group of MHCI experts, based on their past experience and adapting the local emergency plan to the current situation. This group should then report to overall rescue command center as soon as possible. Once permission is granted, these documents should be circulated to all mental health workers and everyone’s understanding of the information should be confirmed, to ensure the MHCI work can progress smoothly.

Another aspect of MHCI that requires attention is clarification of the position of local and external teams. MHCI is a highly district and time sensitive job, therefore, local mental health teams should lead the intervention, with additional secondary support from external mental health teams. Local mental health teams are more familiar with local culture, living habits, and customs, have pre-existing trust, and can communicate more easily with the local community; in contrast, external workers require longer to adapt due to language barriers and other factors. Unfortunately, many regions in China do not have established local mental health crisis teams. We therefore suggest that mental health intervention teams should be rapidly established in specialist psychiatric hospitals, general hospitals or community health services from province to county level, providing a system for MHCI. In the short term, as a minimum, we advise training and managing local MHCI instructors and planning recruitment, training and evaluation for different categories of workers, in order to enable efficient set up of MHCI teams when needed.

There is a consensus on the importance of establishing a long-term tracking and rehabilitation plan for crisis victims, included in the Critical Incident Stress Management (CISM) [23] and Seven-Stage Crisis Intervention Model (R-SSCIM) [24]. In our study, many interviewees were concerned about the lack of follow-up for post-disaster MHCI in China. Currently, no formal work plan and policy are available to guide follow-up mental health rehabilitation. Thus, this study reports recommendations for future management plans: 1) incorporate a guide for follow-up mental health rehabilitation; 2) summarize and analyze information about the important personnel and specific populations relevant post-disaster; 3) build a team specialized for follow-up mental health rehabilitation; 4) continue promoting training and supervision of mental health rehabilitation workers from national to local level.

Our findings also identified ethical problems in crisis fieldwork research. Similar problems have been also identified in Japan; their national guidelines suggest that formal assessments for victims are used to provide screening of high-risk groups and not for research purposes [2]. Researchers should comply with existing professional and scientific ethical principles, and we suggest creating clear principles for disaster research ethics in a national MHCI plan. A working group on mental health and psychosocial support forms part of the 2009 Harvard Humanitarian Action summit, including colleagues from low-resource countries; this working group has outlined key recommendations addressing ethical issues in the conduct of mental health and psychosocial research in humanitarian settings [25], which can serve as a basis for principles included in a national MHCI plan.

Collaborating with media outlets has consistently been a difficulty for MHCI [26]. To maintain a good relationship with media, future plans should include principles for media interactions. Recently, a new study proposed a framework to facilitate the development of disaster social media implementation, identified though a systematic review [27]. We also suggest some additional advice based on our findings. MHCI teams should obey instructions from rescue command centers when communicating with the media and ensure the information provided is accurate and clear. It is critical to avoid any misleading messages which may cause serious public panic or worsen the impact of a disaster; studies found that exposure to the media during the 9/11 terrorist attack was associated with increased risk for post-traumatic stress disorder symptoms [28]. MHCI teams should nominate one to two members of the team as public representatives to be interviewed at press conferences or other media events. Their responses should be comprehensive and accurate and speakers themselves should remain composed. Under all circumstances, the first priority of MHCI workers should be care of disaster victims and protecting clients’ privacy [2, 3]. When collaborating with media colleagues, MHCI workers should aim to understand and respect their work, and guide them in communicating positive messages. Finally, field MHCI experts should make use of all forms of media – including radio, TV, or other new platforms – to disseminate information about available mental health crisis interventions.

### Limitations

This study aimed to include a diverse and large sample of mental health workers with a range of experience, but there may be sampling bias which influenced the results. Additionally, we were not able to control or measure the quality or effectiveness of the participants’ disaster response. Due to the significant importance of authority in Chinese culture, we also observed that in some focus groups the presence of senior workers meant younger mental health workers were reluctant to share their own perceptions, or that experienced/senior workers dominated the conversation. When this occurred, we tried to prompt all participants to engage to ensure a balanced range of perceptions.

### Conclusions

This is the first large-scale research using rigorous qualitative methods to explore the perceptions of mental health crisis intervention held by a diverse range of Chinese mental health crisis workers and government administrators. Drawing on the themes identified through analysis of focus groups and individual interviews, this study provides a new framework for developing national and local post-disaster mental health crisis intervention plans.

## Additional files

Additional file 1: Interview guideline for crisis intervention workers. (DOCX 13 kb)

Additional file 2: Examples and details relating to each theme. (DOCX 85 kb)

## Abbreviations

CISM: Critical Incident Stress Management
MHCI: Mental health crisis intervention
R-SSCIM: Seven-Stage Crisis Intervention Model
TENTS: The European Network for Traumatic Stress
