# Supplementary material for: Mental health workers perceptions of disaster response in China
Source: BMC Public Health. 2019 Jan 3;19:11. doi: 10.1186/s12889-018-6313-9 (PMC6318987; doi:10.1186/s12889-018-6313-9)
Supplement: Supplementary file 1 — Interview guideline for crisis intervention workers. (DOCX 13 kb) [file 12889_2018_6313_MOESM1_ESM.docx]

**Interview Guideline for Crisis Intervention Workers**

1. Could you share your feelings and thoughts about participating in a mental health intervention in a breaking public crisis?

2. Would you mind commenting on the progress of the current mental health intervention?

3. How do you think a mental health intervention should be implemented after a public accident?

4. What do you think are the responsibilities of a mental health intervention worker?

5. What deserves the workers’ attention in mental health intervention?

6. Could you explain how the intervention was delivered in your team?

7. Do you think a mental health intervention after a public event is different from normal psychological counselling or psychological therapy? Why?

8. What are the remaining issues about the current mental health intervention strategy?

9. Could you share the strategies you used on the clients in this psychological intervention?

10. Could you tell us how you apply the psychological techniques you have learned to this psychological intervention?

11. What are the tips you would suggest for applying psychological techniques?

**Interview Guideline for Government administrators**

1. Do you think mental health crisis intervention is important? Why?

2. How do you think we should implement mental health interventions?

3. Who do you think should undertake the mental health intervention?

4. How many people need to be recruited to form a mental health intervention team? And who should be recruited?

5. What resources are required for such a mental health intervention team?

6. Which department and ministries should be called to cooperate with mental health intervention organizations? What kind of team structure should this be? Why?

7. What do you think about the current mental health intervention procedure?

8. How did you organize and coordinate the mental health intervention at the scene?

9. In what ways do you think the mental health intervention is effective?

10. What factors are related to such effects?

11. What problems do you expect mental health interventions to solve?

12. What are the deficits of the current mental health crisis intervention?

13. What information should the mental health intervention organizations provide to the crisis command center? How frequent? In what form?
