# Supplementary material for: Mental health workers perceptions of disaster response in China
Source: BMC Public Health. 2019 Jan 3;19:11. doi: 10.1186/s12889-018-6313-9 (PMC6318987; doi:10.1186/s12889-018-6313-9)
Supplement: Supplementary file 2 — Examples and details relating to each theme. (DOCX 85 kb) [file 12889_2018_6313_MOESM2_ESM.docx]

Catalogue

[1 Hierarchical organization 2](#_Toc503276795)

[1.1 Forms of organization 2](#_Toc503276796)

[1.2 Management system 2](#_Toc503276797)

[1.3 Team construction 3](#_Toc503276798)

[2 Intervention path way 7](#_Toc503276799)

[2.1 Work plan 7](#_Toc503276800)

[2.2 Resource preparation 8](#_Toc503276801)

[2.3 Circular investigation 9](#_Toc503276802)

[2.4 Designated intervention 10](#_Toc503276803)

[2.5 Intervention supervision and training 11](#_Toc503276804)

[2.6 Handover 12](#_Toc503276805)

[3 Intervention strategy and technique 13](#_Toc503276806)

[3.1 Assessment, screening, and referral 13](#_Toc503276807)

[3.2 Workers-client relationship establishment 14](#_Toc503276808)

[3.3 Solving practical problems 15](#_Toc503276809)

[3.4 Psychotropic medication intervention 16](#_Toc503276810)

[3.5 Psychological intervention 18](#_Toc503276811)

[4 Public health information 23](#_Toc503276812)

[4.1 Text-based public health information 23](#_Toc503276813)

[4.2 *Media* interactions 23](#_Toc503276814)

[4.3 Hotline counselling 24](#_Toc503276815)

# 1 Hierarchical organization

## 1.1 Forms of organization

*“After a huge earthquake, I heard that nearly 200 victims needed help at a nearby village. So our team, 10 members or so, went to that village. After we arrived we initially saw nobody; we then started to find people, asking whoever we met. Some said that the victims had been transferred; some said there is no such village at all. We wasted the whole morning struggling around. The problem was that our mental health intervention work did not follow up with the overall rescue work. We were not under the same working arrangement, lacking clear information. As a result, it was impossible to work with good quantity and quality”*

*“Mental health intervention should coordinate with overall rescue work. In mental health intervention fieldwork, many victims, in fact, do not need professional psychological support. Instead, they are dying to know some information, such as where can they get food, where is the tent, where are their family, etc. If we know none of this information, they will be disappointed with us, and even feel antipathy, thinking that we are not helping but making things worse. Therefore, now, before departure for fieldwork, we need to learn about some related information. I think telling them the information they want plays a crucial role in calming victim’s nerves. The premise for obtaining information is a unified organization.”*

*“As a member of a mental health crisis core expert group, one of the important work duties is communicating between leaders and workers. On the one hand, messages from rescue command center need to be delivered to every MHCI member, so that crisis clients can receive the information they care about. This requires me to attend every overall rescue meeting, to keep up with the newest situation and development. On the other hand, I need to report daily performance of the whole mental health intervention team, major problems, difficulties, and the condition and personnel change of the* *mental health intervention team. These two sides consist of a problem-shooting and solving cycle, and this process is done under a unified organization. From a psychological angle, the stability of mental health intervention teams, to some extends, depends on the transfer of accurate information. This also plays a role in the global stability of all victims”*

## 1.2 Management system

*“In my view, the MHCI work of the Wenchuan Earthquake was a complete mess. Everyone was doing their individual job without a holistic work plan or scheme, and no unified management.”*

*“Once we arrived at the Jingzhou, we first went to the local mental health intervention team. Because without their introduction to the characteristics of local residents and situations, we could not carry out our work. We had the experience that we could not find the local mental health intervention team, and our work became very difficult to start with.”*

*“I think the psychological intervention after an accident is different from my normal routine job. Once arriving at the site, I was suddenly dumb, knowing nothing about what I should do. My immediate reaction was to find the organization and accept my task through organization. I felt much more at ease when the rescue command center assigned me to work with the mental health intervention team organized by local medical institution. I could finally carry out my work without worries. Therefore, an organization is of vital importance; it settled me down at the beginning.”*

*“I think in psychological intervention, volunteers cannot only count on our own. You know, there are many things we cannot do. However, honestly, many things still cannot be finished without us volunteers. Therefore, the ideal model is that all rescue forces are organized under a unified organization to work together. These years, during public accident psychological intervention, we are combined with the mental health intervention team appointed by the other government department, e.g. health department, education department. The leader of our volunteers forms a core leading group with the leaders from other mental health intervention teams, and attend the overall rescue meeting discussion together. In this case, we are able to get important information in time, evaluate the current situation, and solve the problems we meet during work. Therefore, personally I would say psychological intervention, in many occasions, requires much administrative side of work.”*

*“Mental health crisis intervention is a district and time restricted work, the local mental health intervention team should be the core force in the overall rescue, and external mental health crisis intervention teams should only support the local team.”*

*“The local intervention team should dominant the fieldwork. As external experts cannot understand our local language, we have to spend too much time translating what clients say to external experts.*

*“I took part in several public accident mental health interventions and had different feelings each time. I think the most important factor for successful mental health intervention fieldwork is whether there is a powerful management organization. If the administrative level becomes paralyzed, then other top-down work will also become a mess. Mental health intervention workers will also feel at loss and be stuck with exhaustion. But if administrative issue is responsive, and the mental health intervention management organization is reasonable, then the overall mental health intervention will produce the best outcome; mental health intervention will always be at a clear stage, and the problems can be solved smoothly. This can maximally avoid work’s powerlessness and exhaustion.”*

*“I think the first task for MHCI is to establish a core group. The members of this group should be able to attend the overall rescue meetings, and even help to make some intervention decisions. In fact, the fundamental purpose is to accelerate and stabilize the stability of overall rescue team, so that the rescue work can steadily progress. There is a psychological principle behind this: because I understand that the accident has a serious impact on the original stability of a certain area or society, just as a baby who is suddenly thrown out from the cradle, entering an erratic condition, full of unsafety and uncertainty. Our task at the moment is not trying to talk to the baby and calm him down. Instead, we need to figure out a plan and strategy, judge the amount and location of the babies, and dispatch a team to bring the cradles and catch up the babies at a fixed position. The logic behind this is that rescue force helps to stabilize, to positively influence the victims, and this is the premise for the relief of individual psychological crisis.”*

*“Our local team should develop a mental health crisis intervention team, it would be too late if the external intervention team takes a long time to come here.”*

## 1.3 Team construction

1.3.1 Team Leader

*“It is undeniable that team work should take priority over individual work, but this might depend on team composition and the quality of team members. Personally I think the leader of the team is very important. He must have the ability to coordinate with different mental health intervention teams, bring everyone together to build mutual understanding and trust. His professional competence must also be first-class, so that everyone can recognize him as our leader, that he can make thoughtful plans.”*

*“Our team leader was great, with strong communication skills. Any problem we met in our work, if reported to him, they would be soon resolved. Our work would meet major obstacles without him.”*

*“Our leader never keeps up appearances, always keeps a low profile. Despite his rich experience, he is never presumptuous; always asks our opinions; discusses with us and then makes decisions. It is delightful to work with him.”*

*“This leader deserves its name. He is indeed our role model. Not only being amazing at work, he also shows perfect personality, with a decent manner of dealing with people. Everything goes on well as long as he is present, and we understand and support each other during group discussions. In fact, to some extent, whether our work can meet the expectations depends on the leader’s encouragement and model effect.”*

*“If there was not an excellent leader whom everyone respected, no one knows how this team could work. It is possible that everyone would ignore each other and everything become a mess.”*

1.3.2 Team Expert

*“What left me with a strong impression is the way that the mental health intervention expert group worked out. In that mental health intervention mission, the psychologists appointed by National Health and Family Planning Commission suggested that local hospitals and university volunteer organization mental health intervention team select several cooperative and professional experts to form an expert group. In that mission, this group played a fundamental role. It gathered the problems we met in work, then offered advice to these problems one by one, and finally adopted the consensus decisions. This also reflected the democratic centralism manner of working.”*

*“Local psychologists know better about their culture, customs, so our external experts should ask for their advice before making any decisions.”*

*“I am grateful to the experts sent out by the National Health and Family Planning Commission, who did not put on airs at all. Although I don’t have much experience in this field, they offered a lot of encouragement and support.”*

*“After the accident, we soon established a psychological intervention team. Although we did not have much experience, external rescue experts have offered much support and encouragement, helping us to make work plans and other mental health intervention materials, taught us how to do intervention from the beginning. This laid solid foundation for later psychological rehabilitation. Therefore, our work became increasingly familiar and proficient.”*

*“During the collaboration with external mental health intervention teams, my largest feeling is their attitude. If they held a condescending attitude, giving orders to us, we would rather they did not come. We found that the more famous the expert is, the more modest as he is. They really helped with us, providing a lot of guidance. We think that these experts not only helped us during the crisis period, but also trained many mental health intervention workers for us, leaving us with much precious experiences.”*

*“I lack the experience of psychological intervention after public accidents. To be honest, I was extremely uncertain when I arrived at the disaster area. I felt I lost the excitement when I first received an order; many worries emerged; and even I fell into crisis status. I was wondering if there would be veteran psychological experts to guide our work. Once arriving at the site, we saw psychological experts from provincial and national level, some of which were the big names we often see in the television and on the newspaper. Their diligent, modest working attitude encouraged us to work more confidently. This experience was unforgettable. Being a young psychologist, I finished my job as well as learned a lot through this experience.”*

*“Psychological intervention covers a wide range of work. Its successful operation involves various factors, which require sophisticated plans during the whole progress. I have attended several psychological interventions, and the most important finding is that mental health intervention calls for a professional, veteran, and insightful psychological expert group. This group, to some extent, determines the efficiency of the whole mental health intervention team. Because the expert group not only has to define the problems in mental health intervention fieldwork, rules out hidden danger, carries on the working plans, but also communicates with upper departments to urge them make the correct decisions.”*

1.3.3 Team Member

*“I remember that we met so many troubles during the MHCI mission in Wenchuan Earthquake. Some victims refused to speak, presenting as quiet and dull. I wanted to approach to them, but they did not respond. I worried about them but did not know what to do.”*

*“After the terrorist attack: in a daily ward round, I found one patient’s mental state was worse than yesterday. I employed some psychological therapies to try to understand and console him, but he seemed absent-minded. After that, I kept thinking that something must have gone wrong, and reported this situation to my team leader. My leader was a psychiatrist. He soon went to the ward to check the ECG after hearing this, which suggested a decrease in oxyhemoglobin saturation. The leader then immediately called up other doctors for comprehensive examinations, and the result was hemopneumothorax. That felt scary even till now! I am so glad that I reported the situation in time, otherwise it might have lead to a disaster.”*

*“I could still remember that when I first joined this work in Jingzhou, it felt uneasy and worrying. I had no previous experience. MHCI fieldwork is completely different from my everyday work. I hope I have a deeper background and more abilities in psychology.”*

*“I entered a resettlement point and visited one family, telling them that we were mental health rescue workers. The response was ‘we don’t know you and don’t need your help.’ This let me down. Later I found a local referee to help us gradually approach them.”*

*“When performing MHCI in disaster area, we first need to find the principle of the resettlement point, introduce us, win their trust, and then ask him to guide us around to see if someone needs help.”*

*“It is important to maintain good cooperation and communication with the principles of temporary resettlement points by flood, hospitals, and schools. Once I sent two MHCI workers to a resettle point to perform intervention. They soon returned because the principal did not allow them to come in. I then personally visited there. At first, the principal also said no. I patiently heard about their concerns, and then expressed our intentions politely, roughly telling him about the procedure and effect of psychological intervention. We then received his attention, and asked him to walk around with us to see how our team would work. Soon he witnessed the effect.”*

*“I was once nominated as MHCI team leader by National Health and Family Planning Commission of the People’s Republic of China (NHFPC) after this plague incident. I think the team composition is of major importance. Being a psychiatrist, I can take charge of the diagnosis of mental disorders and prescription of psychotropic medications. However apparently, MHCI is not psychiatric clinical work, because the majority of the clients are not pathological; they are just crisis clients showing stress reactions. Therefore, a MHCI team must contain workers from other related specializations. There are not many problems purely psychiatric, thus non-psychiatric workers, e.g. psychological worker and social workers should take higher quota.”*

*“Our local Mental health workers lack enough abilities to cope with crisis intervention for the clients. They urgently need further training from an experienced expert.”*

*“The diversity of a MHCI team’s professional emphasis is the premise for its stability. If a team only consists of psychiatrists or only psychological workers, then the psychological intervention might become problematic. People from the same expertise share the same visions. For example, psychiatrists tend to ignore some natural humanistic responses, and mental health worker without psychiatric training may ignore some important medical information. The mental condition of any crisis client is a composition of biological, psychological, and social factors. Therefore, responding to the complexity of humanity, the composition of MHCI should be a multi-disciplinary scientific team.”*

*“The team must be prepared with some background in psychology, otherwise there will be lack of understanding in cooperation, and it easily results in disagreement.”*

*“In an earthquake, I volunteered to join the MHCI team, yet my boss did not authorize that, because I was not in a healthy condition. The situation had not become clear at the disaster area, and the local geographical condition was tough, so he was concerned that I might not be able to take it. Although I was a bit regretful, I accepted his suggestion. I had to be responsible for myself, and should not cause additional burden to the disaster area. It was also possible to engage in MHCI work remotely by telephone, to analyze some situations and guide the operations.”*

*“In the explosion accident, there was this guy who came to the event site on his own. He found me through the overall command center, indicating that he wanted to be a volunteer. Later I learned that he had participated in many other MHCIs, but always on his own, and did not accept any supervision during work. I suspected that there might be defects in his personality. As expected, after greeting with me, he devoted himself to visiting the victim’s family members without any plans. This was definitely problematic. Therefore, I immediately reported this situation to relative departments and personnel to properly handle this person.”*

*“At the beginning of the intervention, we felt that it was very hard to cooperate with psychiatrists. They seemed to look down upon us. I heard in private that they said we only know how to pass over the tissue and talk with the patients. However, they failed to realize the importance of our work, and I think they only know how to prescribe medications. In sum, sometimes, I may feel a little down.”*

*“In mental health rescuing after this terrorist attack accident, my personal impression is that not any single expert can finish this job by themselves. No matter psychiatrists, psychological experts, or volunteers, they all have their own advantages and disadvantages. In order to play the role of psychological rescuing effectively, the cooperation among different disciplines is necessary.”*

*“In my view, the MHCI at Wenchuan Earthquake was a total mess. Everyone was working individually without an overall working plan and scheme, let alone unified management. This is why there was a saying in the disaster area: ‘be aware of fire, thief, and counsellor.’ These years the employment of MHCI has seen much improvement. I think this is because everyone has noticed the importance of team cooperation.”*

*“The development of psychology these years has trained many senior professionals. However, MHCI is different from psychological services; it requires the cooperation among workers from different professions. I have participated several times in MHCI, and am convinced that the condition of a team directly determines the efficiency of the operation.”*

# 2 Intervention pathway

# 2.1 Work plan

*“After the Dongfangzhixing accident, the command from above appointed me as the leader of this MHCI team. I was befuddled at the moment. Although desk work is part of my job at the hospital, I have no previous work experience in MHCI. After calming down, I realized that the hospital office follows various regulations. Workers are required to learn and understand them to adjust their behaviors to a unified order, and then the hospital can work as a whole. Therefore, I think MHCI should first formulate a series of documents relating to the current accident, and all workers, including myself, must follow the regulations set by these documents, to stimulate the operation of MHCI. Fortunately, an expert from NHFPC brought some paper works for past MHCI events. We then seized the time to adapt these documents to the conditions of current accident, and I felt much more relieved. This laid a solid foundation for subsequent fieldwork.”*

*“We need to follow relative requirements and principles in every day psychological counselling and psychotherapies. However, MHCI towards public accident, apparently, calls for special requirements, and we need to be clear about this. Because I understand that the success of MHCI does not only rely on psychological factors, additional factors such as social, humanistic, and even medical, also play a role. Therefore, the formulation of relative documents must be comprehensive. Any missing parts might cause the intervention to become chaos.”*

*“When I first arrived at the epidemic sites, I didn’t know what to do. After carefully reading through the relative documents about this MHCI, I felt much more relaxed. It had been clearly stated what we should do and what we should not, and what were the working forms and procedures, standards for screening and the content to be recorded, etc. Although there was a vague idea about the ideas and principles of psychological work, the formulation of on-site documents surely would clarify our understanding of correct working procedures.”*

*“After I was ordered to participate in the flood rescue, I started to learn about the reports regarding this accident from authoritative media online on my way to the site, and then configured the working scheme. I would refer to past working documents, and made a vague working plan based on current situations, so that I could swiftly establish connections with local MHCI team upon my arrival.”*

*“Clear work plans make our work in perfect order, especially when everyone else is in a chaotic status due to the sudden occurrence of the accident. The stability of the team is even more important than individual psychological intervention skill, and this is dependent on whether we can swiftly formulate a series of clear and operative plans. With clear plans, we can follow this pathway, and feel confident.”*

*“The work requires a plan, knowing what to do and how to do it when. Everything has an expectation. This would be ideal. And it should be clear that this plan is made by group discussion, and everyone should obey to it. Since MHCI team are temporarily established, despite the fact some are from the same institution, they may not work at the same department, so there is no previous collaboration, let alone the title and position are all different. Therefore, a common plan which clarifies everyone’s responsibilities, rights, and duties is necessary.”*

*“Being the leader of the intervention team, I felt under more pressure. My responsibility was to establish a bridge between the crisis intervention decision-makers and the MHCI worker. I needed to make a plan for myself, as well as for other team members, so that everyone could work under regulation without any omission, and saving time for repetitive notifications. This also made the working procedure more standard.”*

*“Once during an accident, our health bureau invited an authority in psychology for a workshop. However the venue was in the city, not at our fieldwork site. We really wanted to attend because that was a precious learning opportunity. Our leader noticed our anticipation, said: ‘I understand your desire for learning. However, we need to be aware the purpose of learning, that is, to better help with the victims. I will send the recordings of that workshop to you later, but right now please focus on our work plans and finish our tasks.’ Upon hearing these words, we then started to realize the importance of plans. Plans indeed can reduce the fickleness and bring more stability.”*

## 2.2 Resource preparation

*“Once after an earthquake, I went to the disaster area with some of my colleagues, trying to work as volunteers. When we got there, we saw many people wandering around like headless chicken. The sky turned dark, we still did not offer any help, and all got hungry. We then had to settle down at a temporary resettlement point, and received some food together with other victims. Even now I feel embarrassed about it.”*

*“Our hospital always puts MHCI at priority level, being prepared for material supply in peacetime, and taking regular check and update. Therefore, once an accident occurs, we are soon able to grab the materials and arrive at the site. Actually, the key to this work lies in daily preparation. Without careful daily treatment, it is impossible to be responsive to emergencies. This substantiates the professionality of the MHCI team of our hospital.”*

*“After setting up an MHCI team, we will appoint a logistical support group specialized for taking care of the team’s accommodation and travelling. Each worker should strictly follow the arrangements made by such group, coming back to the settlement for dining and rest every day after work. The team should not bring more burden to the local area. The supply materials will be imported from outside as required. This operation can provide MHCI workers with basic accommodation, as well as exempt the worries so the team can be dedicated to work.”*

*“I think one of the strengths of the volunteer team is their unified equipment, uniform, name tag, and backpack, which signals an impression of canonicity. You can see that in an event site, although victims did not know their names, they can still find them by recognizing their red jacket in case of any difficulty. Therefore, I think MHCI team could learn from it.”*

*“Being a psychiatrist, the uniform at hospital is the doctor’s white coat. When patients are referred to hospital, I naturally treat them as traditional doctor-patient relations, focusing more on the patient’s symptoms and mental issues. However, in many MHCI fieldwork, when I got dressed as a MHCI workers, I felt that the relation between me and the crisis clients became closer. I paid more attention to the clients as a person. In the meantime, the collaboration with the members from different disciplines also became better. Thus I believe that unified dress code indeed can ameliorate my work.”*

*“It is obligatory to wear a unified hangtag or armband during working. You know, the accident site could be highly disordered. People come and go all the time, and there are people who pretend to be MHCI workers and offer so-called psychological intervention to victims. I had a story like this: in one hospital receiving patients from the accident, several waves of the so-called mental health intervention workers went into a ward. Some encouraged the patients to express their sorrow through crying, some said that they need to hang in there and stay positive to the future. In the end, the patient’s family got annoyed, refusing any visit from any MHCI workers. Obviously, these so-called MHCI workers are not associated with any formal entities and lack of training. They cannot offer any help, and may make things even worse. Therefore, we advise that our MHCI team appointed by the rescue command center must be unified in dress code, wearing hangtags and armbands to work. Wherever we settle down, we need to first get contact with the principles, such as hospital leader, resettlement point principle, etc. Tell them that people without unified markers are not allowed in. Everything must be ordered and scientific.”*

## 2.3 Circular investigation

*“In psychological counselling, there is no such thing as finding the patients. Counsellor always sit in the counselling room, waiting for the patients according to appointments. MHCI, on the other hand, requires workers to be more initiative. To be honest, at event site, even some basic soothing is helpful to the victims.”*

*“Even though daily investigation seems to be not so professional, in fact, it reflects special skills of MHCI workers. That is, MHCI workers must be very sensitive to all kinds of situations, able to capture the major problems and patients accurately, and offer appropriate treatments.”*

*“Circular investigation is like ward round in psychiatric clinical work. It is a daily task, because patient’s situations vary at every day, and we need to ask about their feelings. Of course, crisis clients differ from pathological patients, but in the crisis stage after accident, some of the crisis clients indeed would be in a severe mentally disordered status, which requires immediate response and treatment.”*

*“I think one of the major tasks of circular investigation is to provide crisis clients with some critical information. For example, the location for collecting living supply, location for medical station. Also tell the parents to take good care of their children, elderlies, and other disadvantaged groups.”*

*“The circular intervention in my definition is visiting the victims with a helpful attitude as a professional, detecting and solving the problems. In problem solving, we should not show off our expertise, only focusing on our job as if providing counselling sessions in clinic room. Instead, we should offer some practical support, referring to other treatments when necessary.”*

*“Once when I knocked on the door of this family and introduced ourselves to them, they treated us with bad attitudes, saying ‘get out, get out, we don’t need your help.’ We felt very uncomfortable, but our leader told us that this was the meaning of our work. While observing the way people communicate with us, we could learn that how this accident influenced this family. Therefore, we must show our tolerance and understanding.”*

*“When visiting the victims, some victims often grabbed my hand, talking about their difficulties, crying. In this situation, I would look at them with compassionate, and listen to their confession. I would tell them, we have many other people who would help them to get through the difficult times, and told them we would come and see them again when leaving.”*

*“Circular investigation plays an important role in MHCI. It is not only about detecting problems in time and solving practical problems for victims; from a social psychology perspective, it also enacts a supportive, steady influence on the group mind. When MHCI workers first enter the site, maybe some victims will be susceptible, and even in some cases, some local residents will pour out their anger and complaints onto MHCI workers. However, MHCI team’s steady and standard working procedures will placate these victims and change their attitude towards outer environment and other people. This, in fact, is the process of social stabilization.”*

## 2.4 Designated intervention

*“Circular investigation and designated intervention together contribute to a satisfactory MHCI work mode. We arrive at the flood place, and visit the families one by one. Whenever we spot any emotionally unsteady client, we find more about him through observation or chatting with his family, and report to the leader. The leader then collects the information and reports to core expert group. Finally we figure out an individual intervention scheme: whether referring to mental health institutions, or sending worker for continuous designated intervention sessions.”*

*“In this explosion accident, one nurse witnessed the bleeding corpses being transferred to the hospital. Since that day, she felt too scared to stay at home alone, with all the flashbacks of those horrible scenes. She then came to us for help, to get rid of these horrors. One of the trauma therapists in our team offered her EMDR sessions, which was very effective.”*

*“In one investigation task, we found someone with abnormal behaviors at the resettlement point, with salient depressed mood and glazed eyes. We then entered his house and found a rope. We immediately got in contact with his family and knew that he once suffered from bipolar disorder. According to the psychiatric assessment this time, there is major depression going on, and we thought he was of high suicidal risk. After patient communication with his family, he was referred to local mental health center and taken care of.”*

*“One policeman was injured in this terrorist attack, and was sent to the hospital. The nurse reported that he slept very badly, even with screaming and shouting. We realized that this policeman must be in severe stress status. However when we asked about his situation during investigation, he always presented as calm, saying that he was fine. We thought that he must have been embarrassed to ask for MHCI team’s help. Based on this condition, I told him straightly about other people’s psychological response against public accident, and promised that anything he said would be absolutely confidential, and then he agreed to cooperate with us.”*

*“Once an old gentleman who lost his wife in the Dongfangzhixing accident complained to me that he had an appointment with a MHCI member at 9 am, but the member did not show up until 10. I could see his disappointment and anger, and apologized to him. I then asked more about this case and answered some of his questions. Later, I found that member and reiterated the importance of work setting, making him realize that a stable setting is the guarantee to reestablish a crisis client’s sense of safety.”*

*“I should always be aware that MHCI cannot solve every problem. Therefore, after I establish designated intervention cooperation relation with a client, I will put the focus on his current mental state and use some strategies to make him feel better. We should not probe their past experiences. If the client is in critical condition, and will need some time to recover, I would explain his situation to his family, and offer some advice.”*

*“In one designated intervention session, the client kept describing the horrifying scenes at the accident. Even I became a little scared after hearing that, starting to imagine those bleeding scenes involuntarily. I suddenly realized that I might be led into it. So after expressing my understanding and sympathy to his horror feelings, I asked about how he was rescued under such condition. Then he started to talk about his experience of being rescued……”*

## 2.5 Intervention supervision and training

*“Daily meeting is necessary. In these meetings, experts can offer advice on the difficulties I met in work, and some practical problems can be directly reported. If the officials are present, these problems might be solved right away. For examples, some principals of the resettlement point are not familiar with our job and did not allow us to communicate with the residents. This problem could then be solved simply by a phone call from the official. Of course, for me, the regular meetings bring me a sense of warmness and support. Initially, I really did not know how to make duty reports about my job situations, yet the psychological experts know how to respect me, encouraging me to say more, and patiently helping us to analyze the problems. Therefore during regular meetings, I received not only knowledge and skills, but also understanding and support. This becomes the motivation for me to finish my MHCI responsibilities.”*

*“I think regular meetings resembles group supervision. The posture I present in the meetings, to some extends, exerts an influence on their subsequent working condition. Compared to the problems they reported, what I care more about is their status. Acknowledgement should be granted to their achievements to help strengthen their confidence. As for their shortcomings, I should offer abundant analysis and explanation to help them learn from failures. During this process, in most times I remain as a listener. On the one hand this is to understand the situation, on the other hand MHCI team members can release some stress and anxiety while talking. After that, conversely, I answer some of the questions, and offer some support, to enhance mutual recognition and trust.”*

*“Actually, initially, I didn’t care much for regular meetings. After a whole day’s work, all I wanted to do is to have a rest. Also I thought there is nothing wrong with my duties, so no need to report again. It’s a waste of time. However, the meetings are required by the expert group, so I have to attend. After attending, I realize that it is worth doing. First, I find that there are some minor deficiencies I can improve, thanks to observations from other colleagues. Second, everyone possesses some values which I could learn from, and it is an enjoyable experience to share my opinions with the team. In sum, the meeting process is a place to share knowledge and offer mutual encouragement.”*

*“I received short training in mental health crisis intervention at the beginning of work. This indeed was helpful, as it clarified many of my questions and relieved my anxiety. After all, MHCI differs in many ways from my daily job. In the meantime, I wish the training could involve more case analysis and role playing. Maybe this would help me to better understand the principles. In addition, I think training should be divided into stages. At the initial stage, just telling us what is correct and what is wrong. Later we can include deeper discussion and training according to the developmental pattern of crisis client’s mental state. At the final stage the training could tell us about sadness and trauma treatment.”*

*“Training must be in accordance with the properties of the accident and the needs of the victims. All in all, training should start from public requirements, not be designed for MHCI workers. All training must be done on the basis of not interfering with normal MHCI operation. Important issues and techniques must be stressed, since this should not be treated as free training sessions for MHCI workers. Content such as ‘psychiatric analytical therapy’, ‘couple therapy’, and ‘family therapy’ should be avoided. The important skills should include ‘psychological first-aid’, ‘stabilization technique’, and ‘grief counselling’, etc.”*

*“Supervision is so important for me. In some cases, I have no idea about some situations, even though the training process has provided me with some relative knowledge and skill. However, when being immersed into that environment, seeing those people who lost their family, suffering from great agony, I really felt depressed. I wanted to help but kept doubting whether I was able to. Thanks to the supervision system of my MHCI team, some problems could be discussed at group supervision or individual supervision - this not only clarified some complex issues for me, but also relieved my negative emotions.”*

*“I once was dispatched to console the family of the dead at a mortuary house. However, when I arrived and heard the crying, I suddenly could not move forward, feeling extremely scared and painful. I could no longer stay at that place so I went back. After that I felt so embarrassed, not brave and solid enough, and failed to finish my job. Then a professor from the expert group found me, made an appointment in a quiet room to talk about my experience at that day. When I saw his patient and warm face, staring at me, I was impressed and started crying. I remembered my grandma who had just passed away……”*

## 2.6 Handover

*“One of the most memorable lessons I learnt from MHCI is that things become increasingly difficult as the time goes on, sometimes it might turn into guilty feeling. Because from the perspective of psychology, some crisis clients are still in an unstable status, indicating potential risks for future mental disorders. As the overall rescue work has come to an end, however, there is no subsequent psychological rehabilitation scheme for these clients. Although we try to do something; there seems to be no alternative ways.”*

*“After this accident, we made a psychological rehabilitation plan for crisis clients at later stage of MHCI. However things were more difficult than we expected, since there were no clear rules and requirements on relative training and implementation of the plan. The personnel of our MHCI team were not reassigned to crisis client’s psychological rehabilitation plan, handing over this job ponderously to the lower mental health institute. Obviously the quality cannot be guaranteed.”*

*“From my practical experience about psychological therapy, many patients once experienced major traumas in their life, however, unfortunately, they did not receive help and support in time after that. As a result, the difficulty of treatment increases as time goes by. Similarly, we need to notice that if we do not offer subsequent support for those crisis clients after this public accident, their inner trauma may continuously affect their future life.”*

*“I think the follow-up work of MHCI is as important as the emergent stage. Yet due to many internal and external factors, this duty has not received enough attention, therefore its implementation is not ideal. In fact, the follow-up of MHCI involves various types of work, for example the trauma therapy for crisis clients, scientific research on group disaster community rehabilitation, and policy research on future MHCI management, ect.”*

*“Crisis is the combination of danger and opportunity. During public accidents, many opportunities occur in rehabilitation stage. As for individual patients, if they could receive professional assistance at this moment, then not only could they get rid of the crisis status, but also reach for a higher benchmark in their life. As for community, if policy support for subsequent psychological rehabilitation is available, the result would be more than the healing of public mental health, it is also beneficial to the cohesion of the community. As for government, research on post-accident follow-ups can provide abundant cases for future MHCI implementation.”*

# 3 Intervention strategy and technique

## 3.1 Assessment, screening, and referral

*“I am strongly against disseminating questionnaires to crisis clients after an accident, especially I have an aversion to the act of ignoring client’s feelings during the questionnaire circulating process. This will cause severe damage to the clients, and also challenges the position and principles of MHCI.”*

*“In my investigation session, I never use questionnaires and ask on behalf of clients, since this might lead our communication to become stiff. Many important responses from clients will be missing, also they might be suspicious about my purpose, losing trust towards me. If thinking from their position, I might think taking a questionnaire is just a way to use my agony to satisfy their need for scientific research, which is unethical.”*

*“I used to treat assessment and screening as the same procedure, but now I believe that these two should be separated. Screening is mainly for solving the problems at present, ensuring the maximal interests of the crisis clients are protected. However, assessment is of equal importance. Because only through in-depth, careful, and comprehensive research can we accumulate more experience and knowledge to cope with similar situations in the future.”*

*“We met with serious trouble once during the MHCI work after the shipwreck. One lady’s husband died in this accident. Upon hearing this news, this lady presented with serious psychotic responses, claiming that her husband was waving his hand towards her on a small island far away, and she could hear his shouting at her. This was typical illusion and delusion. After careful consideration, we made the decision to refer this lady to a mental health institute for further psychiatric diagnosis and treatment.”*

*“The majority of the victim clients can recover to normal life and work on their own ways. Therefore, MHCI workers should avoid over-intervention. That is, apart from those clients who require further designated intervention and referrals according to the results of screening, most victims only need the assistance from circular investigation.”*

*“For some of the crisis clients, we cannot offer a conclusion on whether his condition will get better or worse based merely on initial screening. Therefore, we will make appointments with them according to their situations. In this process, we are trying to improve their social support, observe their behaviors, and judge the degree of trust between us. Only after gathering necessary information can we decide further operations.”*

*“Questionnaire assessment has always been a debatable issue in MHCI, some think that questionnaires are not applicable in MHCI, while other believe that questionnaires are a useful way to know more about client’s situations. However, I think if only MHCI workers could handle it with flexibility, questionnaire should not be a problem. As long as MHCI workers put crisis client’s interests as the first priority, questionnaire can be implemented in a good way.”*

*“Not all clients are suitable for interventional research, they should be evaluated carefully before starting the research.”*

*“Always, we could not force them to participate in the research, which should be based on crisis client’s permission.”*

*“MHCI workers must show comprehensive qualities in their MHCI fieldwork, that is, besides the correct method, humanistic care is also necessary. For example, in one MHCI we participated in, there was another team with us. When meeting with the clients, they simply put out the questionnaires and asked them to fill in, and after that, they only said ‘thank you’ and left already. This was indeed outrageous. Although taking questionnaires showed their prudence in work, lacking of humanistic care should still be considered as unethical.”*

## 3.2 workers-client relationship establishment

*“I think in MHCI, the most significant part is how to establish a good relationship with the clients at the beginning. To make a vivid metaphor, after the impact of an accident, clients have suddenly returned back to little babies, at which point they require a bolster. In the meantime, MHCI workers were like mothers who need to accompany with these babies.”*

*“When first approaching the clients, the first priority is to win their trust. I understand that to achieve this goal its not simply with words, non-verbal behaviors and attitudes are more effective. For example, when clients are relieving their emotions, we need to stay by their sides and keep reminding them that we are safe now, ensuring them that we can help you. Remember the phrase ‘more work less talk’, because for people in crisis status, excessive talking is just noise.”*

*“I think we, the volunteer group contribute a crucial part to overall rescue force. After standard training, we play an important role in the rescue work, especially during the initial stage to establish contact with crisis clients. Our passion and empathy, and the dedication to solve practical problems for them will make crisis clients feel warm, respected, and well understood.”*

*“To tell the truth, most clients I engage with will gradually recover to rational and peaceful status. As our mutual understanding moves forward, they changed from being initial passive and helpless to initiating talking with us, and started to plan for the future. Now, being MHCI workers, not only should we continue our support, but also show full respect to these clients. We need to think twice about our positions and realize that we are just a supportive role, so that we can further improve crisis client’s initiative.”*

*“If the relationship between crisis clients and MHCI workers was like a baby to their mother at the initial crisis stage, then MHCI workers should adjust their role accordingly when crisis clients start to regain their initiative. Now MHCI workers should be like a father, who believe that their kids can walk out from the adversity on their own way. Support and encouragement should be the main point.”*

*“Once we were ordered by the government to take care of several injured living people. At the beginning, we fed them food and water, and washed their feet. Later when they got better, they were able to eat and walk by themselves. However, the higher level still instructed us to serve them as before, and we felt increasingly uncomfortable: some chores, which could definitely be handled by themselves or their family, were pushed to us, and they even came up with more and more requirements.”*

*“At this stage, I think besides maintaining cooperative relationship with individual clients, it is important to know more about the client’s family and interpersonal relations, digging for crisis client’s positive interpersonal resources to gradually transfer this relationship to other people who can provide them with sense of stability and safety within a community.”*

*“Termination of MHCI means saying goodbye to the clients I have been helping with, which is very depressing. I can also feel their reluctance to say farewell. However, I have to retain a professional attitude and position. I know that this is my job, to help my clients with their anxiety for saying goodbye. I need to make them realize how amazing their behaviors are since the accident occured. They have devoted very much for both themselves and others. Though there are difficulties awaiting them, there is always a bright future for them.”*

*“The woman I took care of lost her daughter in that shipwreck accident. My age resembled her daughter’s, thus she was extremely dependent on me. I did not know how to tell her that our duty was about to finish. Later I gave her my phone number, but I didn’t expect that she would call me day and night. Although I tried very hard to answer her calls, but, you know, I am not allowed to use phone during work. I had to refer to a volunteer association, hoping they could arrange a psychological rehabilitation plan for this lady.”*

*“Being a MHCI worker who has attended several MHCI after public accidents, I still need to emphasize that every working experience is a baptism of my mind. I learned a lot from those crisis clients. I was impressed by their tenacity and unity. Therefore, before I left them, I sincerely expressed my gratefulness, and sent them best wishes.”*

## 3.3 Solving practical problems

*“To tell the truth, I had some false understanding of MHCI. I thought that mental health workers should focus on psychology; as for the practical problems from the victims, those should be taken care of by volunteers and social workers. However, being a MHCI worker for a longer and longer time, I gradually realized that this is wrong. Humans are a composition of biological, social, and mental factors, the help we offer to those victims should also show this composition. If we only help them using one identity, then the results would never be ideal.”*

*“Social work is my major. I remember that I was very confused during my first experience of MHCI. I admired my colleague psychiatrists and psychologists, who could make use of their expertise, while my responsibilities seemed not important at all. The leader of our team seemed to realize my depression, saying ‘Every single MHCI worker plays his or her role. As for a worker with a social work background, you should substantially fulfill your duties, which are helping victims to solve their practical problems. This requires professional knowledge, and is never easy-peasy.”*

*“As a psychiatrist, we share some advantage in practical MHCI work. Because, you know, the most important responsibility for clinical work is to guarantee patient’s life safety. Therefore, in MHCI, we are always cautious about this, observing closely the mental conditions of the patients to see if they are related to their injuries. Once potential risk has been spotted, we will handle it immediately. I think this is an important part of crisis intervention.”*

*“When I was on my duty of the psychological rescuing at Wenchuan Earthquake, the most impressionable memory was a policewoman breast-feeding the baby victims. That scene was so touching. In fact, during our work, we usually ignore the group who could not directly express their needs. This, exactly, reflects the attainment of a MHCI workers.”*

*“What exactly needed to be done differs from person to person. In different situations, I need to treat them flexibly. For example, for some senior adults, I take good care with their health condition, asking them if they feel uncomfortable frequently, and refer them to the doctors in time. When serving some families, I will take care of the kids, playing with them so that the adults in the family could save some time for other emergencies. Even though these jobs seem simple, I feel that this is what the victims need the most.”*

## 3.4 Psychotropic medication intervention

*“Although the majority hold that crisis client’s mood swings do not require medication, I think this does not cover for all situations. For example, three days after the accident, some people still cannot have a good night sleep, even though they have been settled at a safe place. Therefore, medication treatment is necessary, but must be assessed by psychiatrist.”*

*“The medication treatment in MHCI clearly differs from psychiatric clinical treatment. Although the dose of the medication in the overall working period is not large, once there is a mistake on the medication, serious consequences might occur. Therefore, I suppose that we must take care of every single detail of medication treatment. For example, specifically recording the medication and personnel, make sure everything is safe.”*

*“Having been involved in many MHCI works, the importance of psychology becomes increasingly salient, and I also realize that the comprehensive ability of a psychiatrist is the premise for successful MHCI treatment. Before I was accustomed to paying more attention to patient’s symptoms due to my profession, therefore, in the disaster area, once I saw the unstable crisis clients, I started to think about what medication should be prescribed. However, when I saw other MHCI workers adopting non-medication treatments and also receiving good results, helping crisis clients to recover, I felt I learned a lot.”*

*“In my daily job of MHCI, I hear a lot that crisis clients complain that they were not able to fall asleep for several consecutive nights, and being fatigued at day time. I would advise him to take some sleeping pills before going to sleep in short term. He agreed, and it was indeed helpful. Of course I told him that he could not rely on medication for sleeping.”*

*“Despite the fact that I was strongly against using psychotropic medication in MHCI at the beginning, my thoughts have changed in recent years. Because we need to treat crisis clients from an integrated perspective. Not only should we understand the huge impact the accident has brought to the client, but also we need to know his or her personality. In the meantime, we also need to keep an eye on potential psychotic symptoms. Therefore, if the psychotic symptom cannot be explained by his personality or by this accident, or this symptom cannot be handled by short-term psychological intervention, then medication treatment becomes necessary. The medication could be an anti-depressant, anxiolytic, etc. The details should be referred to psychiatrists, since I am a psychological practitioner. However, still, as for me, at least the crisis clients should be referred for assessment for medication.”*

*“Sometimes, people would tell me that their relatives cannot sleep well for a couple consecutive days. We thought that some sleeping pills might be helpful with their sleep. However, they worried about if the medication was going to be addictive. After our detailed explanation, they finally became convinced.”*

*“There was one woman who lost his son in that explosion accident, and she started to experience illusion, delusion, and insomnia. We escorted her to the hospital for treatment. Later, psychiatrists prescribed her some Quetiapine (an anti-psychotics), and the woman began sleeping all day. Her family started to worry and asked us what happened. One psychological expert, also the director of psychiatric department, suggested that she was overdosed. The dose should be cut half. Because that condition is not a psychotic illness in common clinical behaviors, the crisis client could also not eat and sleep. This physical condition rendered low medication tolerance.”*

*“Once in an investigation, we found a client carrying a big bag full of medications. After gently approaching and getting to know her situations, we learned that she lost her daughter in this accident, so she came here with other family members to deal with her daughter’s aftermath. She had been suffering from mental diseases for years, feeling depressed and light sleeping. The hospital of her hometown prescribed too much medication, for tranquilizers there were several kinds, and she was taking them all. She seemed sad when talking to us, but the overall state was fine. After reporting her situation to the expert group, suggestions from the experts were not adjusting her medication at the moment. Instead we should put her under careful observation, and referred her medication reactions to the experts at her place to provide subsequent medical care.”*

## 3.5 Psychological intervention

3.5.1 Mood stabilizing technique

*“A stable emotional status is the most important thing for crisis clients after public accident. However, this goal is not easy to achieve at the beginning, and neither it is a practical goal. The victims I witnessed are usually either crying, or terrified, or completely silent, or showing impulsive behaviors. We must stay calm in these messy situations. Do not try to persuade them to do anything, instead we should accompany them, staying by their sides. Using their body reaction to understand their feelings, and using practical actions to protect, support them. Because when people are involved in the emotional waves caused by natural disasters, our work at cognitive level, e.g. persuasion, explanation, will be futile. It is malposed communication.”*

*“In fact, in some cases, when faced with crashed crisis clients, I also feel hopeless, not knowing what to do. I am also filled with strong sadness and impotence. However, my supervisor told me that, at this moment, my inner feeling is coherent with what crisis clients feel, and the way to help me regain inner stability would be the best assistance for crisis clients. Therefore, I need to adjust myself, or seek help for my inner crisis status, and then present in front of crisis clients with a stable mind.”*

*“A woman lost her husband in a major accident, and this bad news rendered her in psychotic condition. She was then sent to the hospital for treatment. Later, this woman’s brother came from the other city. We thought he would help dealing with the situation. Instead of our expectation, he fainted right away after arriving at the site. At this time, one post-accident rescue team worker stepped forward and asked him to behave like a man. This young man suddenly got up and intended to hit on this worker. I saw people starting to run away from this drama, only my team leader walked towards this angry man. We were so nervous about our team leader, worried if he might get hurt. The man did not attack. Under our team leader’s staring, this man put his hands on our leader’s shoulders and started crying like a little baby.”*

*“At the very beginning of MHCI, I keep noticing myself not talking too much. If crisis clients start to talk, we should just listen while observing his response and change continuously, and offer some concise, effective, and accurate responses in time. I find that by doing so, they calm down while talking. I suppose this is the power of listening.”*

*“Sometimes, I find that crisis clients remain extremely silent after confronting the disaster. I walk up to them. If they do not resist me, I sit beside them quietly. Later, I gently ask them do they want to talk, and suggest that it will feel better if they can say something. Some of them may respond, and start to express their agony, sadness, worries, and guilt. Now, my job is to handle these emotions and feelings.”*

*“How well a MHCI workers can listen to client’s talking reflects his or her professional level. For those workers who lack sufficient work experience, when facing a crisis client in adversity, they often tend to hurry things up, hoping to solve the problem as soon as possible. Therefore, guided by this attitude, they might offer too much explanation and consolation when crisis clients are still feeling depressed. Clearly this would not be helpful. It is useless to client’s mood stability.”*

*“When I first attend MHCI work, I hated dealing with the occasions where clients lose their controls. I didn’t know what to do. Later, after receiving some supervision and training, I felt that I can influence crisis client’s negative moods, and made adjustments about that. However, still I felt strongly helpless sometimes. I think it is not enough to just listen and understand about clients’ situations. Additional strategies and techniques that are more proactive are required to stabilize crisis clients’ moods.”*

*“Sometimes I think stabilizing crisis client’s mood is like taking care of a little baby. If a baby is crying, I would first check is he hungry? Urinating? Or any other uncomfortable situation? If one of these happened, I will soon solve the problem. If not, I will cuddle him, stroking him. If continuing to make a loud noise, I stroke him more frequently, moving my body around to amuse him and distract his attention. Then, I gradually slow things down, and he becomes quiet immediately. Thus, I think as a MHCI workers, when we spot some crisis clients with dramatic emotional swings, we need to search for a status that resembles theirs to resonate with them. Then, we change our status gradually to lead them onto a more stable status.”*

*“I think regarding crisis client’s unsteady moods, MHCI workers should adopt a step-by-step form of intervention. Under some special circumstances, immediate intervention should be operated on clients, without harming their interests. For example, when crisis clients are consumingly describing their terrifying experiences, MHCI workers should not let them be immersed in such a mood for a long time. Workers ought to provide clear and accurate information during the gaps of their speech, telling them that you are safe now, and right now we can do some activities to placate these horrible emotions. If they do not finish saying everything, there will be opportunities for them to talk about that in the future. This method does not indicate that worker do not respect or understand crisis client’s emotional expression. This is to help clients to realize that they are able to get rid of their negative moods on their own.”*

*“I have a thought on the intervention into crisis client’s anger emotion. This differs from other emotional states like agony, sadness, or desperation. If a MHCI worker says something like ‘it is obvious that you are very angry right now’ to the clients who are expressing their anger, this empathic expression cannot help to reduce crisis client’s anger. On the contrary, it may exacerbate their anger emotions. Therefore, when dealing with angry clients, MHCI workers need to see through what is behind their surface anger, and express their understanding and empathy of this hidden emotion. For example, a worker could say something like this to angry crisis clients: ‘This is just too much for you. Nothing could make you feel more miserable.”*

3.5.2 Cognitive integration technique

*“I notice that crisis clients are not able to talk about the complete picture of their accident experiences. They tend to exaggerate the horrible scenes they witnessed and their helplessness under that condition. In fact, many positive elements in the accident are missing. These positive elements include not only crisis client’s calmness and persistence during crisis, but also the support and help they receive from others. Therefore, during intervention, what I need to do is to restore this content at a cognitive level, diluting their negative thoughts.”*

*“Once I met this survivor of the flood. He endlessly talked about the experience of him being flushed away by the flood, crying. Looking at his desperate and hopeless manner, I asked about how he was rescued. He stopped a while, and started to say how he was flushed beside a rock. He grabbed that rock tightly, shouting out for help. Later, he felt a pair of strong arms dragging him out of the water. He rose up his head, finding out it was the secretary of his village.”*

*“I read much about the literature in MHCI, felt that I am equipped with some principles, strategies, and techniques of MHCI. However, when standing in the disaster site, my mind turned blank. Or, I felt that the words I say to crisis clients were not actually helpful at all. Later, continuous learning, training, and supervision told me that MHCI fieldwork should never turn rigid and dogmatic. Never treat crisis clients as objects for psychological intervention treatment. In turn, we need first feel what crisis clients feel, express care, understanding, support, and encouragement in a natural way. In sum, there is one principle to follow, that is, in the conversations, the attitude should be positive and affirmative, invoking crisis client’s independence and initiative.”*

*“I totally understand all the ideas people might come up with after experiencing this terrorist attack. In fact, this is the normal response under emergencies. However, if these thoughts last for a long time in crisis client’s mind, then it clearly will affect their recovery and social functions. Therefore, MHCI workers should intervene in these situations. First, I need to know and understand what kind of deviations they have, and then use a neutral and peaceful attitude and more objective languages to modify their thoughts.”*

*“When I first attended MHCI, I would feel up and down when crisis clients were talking about their experiences. I often felt speechless, not knowing what to do. Through some training and learning I realized that it is crisis clients’ paranoid thoughts that cause my mind to be unstable. What I should do in such a case is to find out if the client sees the part of their own, of others, and of the outside, and then give them feedback calmly and patiently.”*

*“I participated in the training for narrative therapy, which, I think, could be very useful in MHCI process. The core techniques of narrative therapy, such as story-telling – reorganize and interpret the story, problem-externalization – separating problems from person, and from-thin-to-thick – forming a positive and strong self-image, these techniques can all be incorporated with rapid and effective MHCI fieldwork.”*

*“During MHCI, sometimes I feel that crisis clients are not receiving help from me; instead, they are actually helping me. If we think about it, if I was faced with their conditions, how would I behave? Would I be stronger, more optimistic than them? Therefore, when I see them stepping forward in horror and agony, my heart is full of respect towards them. I would express my respect to crisis clients straight-forwardly. I wondered what belief encouraged them to hold on, and some of their answers impressed me.”*

*“I have paid much in my years of MHCI work. Yet I think what I learned from those experience is much more than what I paid for. The lessons I learned are the enlightenments and thoughts on the meaning of life and human’s love for each other provoked by those victims in the disaster. Therefore, every time I participate in MHCI, no matter how difficult the crisis client’s condition is, I always believe that they will think about this disaster, and even obtain some new understandings about life. I expect them to share their thoughts with me; I am always listening.”*

3.5.3 Behavioral leading technique

*“Relaxation techniques are frequently adopted in MHCI. Because MHCI workers will often need to deal with crisis clients who present with tense emotions or sleeping disorders after accidents. However, many worker are rather rigid and stiff when performing this technique, ignoring the principles and procedures. This has resulted in poor efficiency of the technique and even some side problems. Therefore, the implementation must be based on crisis client’s absolute trust in MHCI workers, and the worker should be wary about the opportunity for implementation, following the principles and correct procedures.”*

*“In crisis situations, it is difficult for crisis clients to find memories that can help them to relax. In any case, we need to believe that crisis clients will use their original positive resources to get out from trauma. It is essential to lead them to retreat to their beautiful past memories at proper times, so that they can be in a relatively relaxed status. Because extracting beautiful memories is a way of relating the past to the future.”*

*“Breathing relaxation techniques look simple, its real operation is not that easy. I once performed breathing relaxation on myself, and it took a while to become accustomed to this relaxing rhythm of breathing, let alone those crisis clients. Therefore, when performing breathing relaxation with others, I am more patient and meticulous. I even demonstrate to the clients several times, and then ask them to follow me.”*

*“Implantation of positive resources plays a crucial role in MHCI. Take as an example, just when we feel tired during work. We must first eat enough food, drink enough water, and then move on to work, instead of working with fatigue. Therefore, sometimes, some MHCI workers only pay attention to reducing crisis client’s negative emotions, while ignoring ways to provoke their self-inner forces. This may lead to poor efficiency of the intervention. Not being able to relieve their negative emotions swiftly, crisis clients might be frustrated, and then more depressed.”*

*“After the occurrence of disaster, it is as if crisis clients suddenly fall into a mire, preoccupied by all sorts of negative emotions, feelings, and cognitions which paralyze and even suffocate them. At this moment, MHCI workers should not jump into the mire and try to drag crisis clients out from it. Because this move cannot save them, and more seriously it is possible to sink into the mire together with them. What MHCI workers need to do is to throw out a life buoy, making sure that they hold tight to it and do not further sink down. And then, provide them food, water, and other nutrition, waiting for crisis clients to be fully loaded with energy and pull the string to drag them to the side. This is my understanding of the procedure for positive resources implantation technique.”*

*“I learned about the safe box technique in some training sessions. However, in my first intervention job, things were not that smooth. Later, I came to realize that the implementation of techniques should be based on cultural background, personal characteristics, the severity of mental condition, and other factors, it is especially important to explain the purpose of this technique at the very beginning. Now the expected efficiency can be achieved. Crisis clients now feel that they are more able to control their unsteady state. The technique can reduce the degree negative emotions affect their normal daily life. Also, when communicating with MHCI workers or future psychological therapists, they can reveal more about their real feelings, and so handle these emotions more effectively.”*

*“Whether a psychological intervention technique can be fully effective depends on many factors such as crisis client’s personality. For example, when applying safety island, some clients may be more likely to follow my guidance and finish the procedures. They also feel very well after one session, and later they can apply this technique in daily life. However, some other clients might find it hard to be involved in the situation. Therefore, as for me, one single technique is not enough to help clients. We should integrate all kinds of techniques to help with clients’ problems.”*

*“Separation is hard for anyone. However this is something we have to face to, because every separation means a new start, and also could be a new opportunity to grow. In my clinical work, the root problem of most patients is that the grief of some separation has not been properly dealt with, and thus does not go out from the depression or anger as soon as possible. Some of them even change their personality. Therefore, in MHCI, I adopt some special ways to help deal with the separation crisis clients experience according to their unique situations. Though this may seem painful and even cruel at the moment, for the long term clients will return to reality sooner, regain their life balance, and even move towards a higher level.”*

*“Separation technique such as writing a farewell letter, I use this sort of technique very carefully in MHCI. Before adopting this technique, I must be 100 percent sure that the crisis client has obtained enough energy or social support to attend a farewell ritual for their lost beloved ones. Therefore psychological stabilizing techniques such as positive resource implantation should be implemented right after the accident.”*

*“The implementation of MHCI must respect local cultural background. I remember that once an earthquake happened in an ethnic minority area. The rescue command center required our MHCI team to perform psychological interventions with the victims. However, after fieldwork interviews, we found that most local residents were religious. They used traditional customs to express their mourning to the dead. After this ritual, they appeared very peaceful. So we reported what we saw to the rescue command center, saying that the local residents had their own way of mental recovery. Therefore, we suggested do not push additional interventions instead of their own way of handling grief.”*

*“I still remember that, in the training sessions, teacher mentioned that if crisis clients show nervous and terrifying reactions to past traumas, and traumatic scenes keep going on in their mind, then we can adopt desensitization therapy, e.g. systematic desensitization therapy and exposure therapy. However, I always feel that these techniques are not very suitable for a crisis situation. Later, I attended EMDR training. I found that some of the elements can be incorporated into intervention. For example, using EMDR on crisis clients with simpler trauma. The result is very promising.”*

*“I often use EMDR technique in rescue work. I remember that in the shipwrecks, an epidemic prevention worker who was responsible for the sterilization of dead bodies started to experience traumatic flashback and nightmares, which had seriously affected his work and living status. Therefore, we performed EDMR on him, and received good effect.”*

# 4 Public health information

## 4.1 Text-based public health information

*“I always bring the mental health brochure I use in the hospital everyday with me before departure. These brochures show the content of MHCI. However when we arrive at the site, we will adjust the content as per current situation, and liaise with relative departments to print and disseminate them. Specific content will include the common manifestations of mental crisis, tips for treating mental crisis, etc. It should also include the telephone number of MHCI hotlines. The dissemination should be incorporated into the whole MHCI work. Not just handing out, we also need to provide some explanation. Our primary audience should be the middle-aged, asking them to take care of their kids and elderly relatives. This job is of much importance.”*

*“Four cornerstones of health education are healthy diet, regular exercise, limiting cigarettes and alcohol, and mental harmony. These tips should be remembered, because after the crisis, many victims lose their regular life and eating habit. The loss of finance or of family members drives them into deep agony and uneasy. Some might drown their worries in drink, and be in a mentally uncontrolled and unbalanced state. Therefore, every one of our MHCI team should hold a general idea about health, offering health education properly, since this is the premise to help crisis clients to recover to a normal state soon.”*

## 4.2 *Media* interactions

*“To be completely honest, I have an aversion to the media. In some cases, some media attend our meetings without any prior notice. Since the meeting will be discussing some issues of personal privacy and uncertain decisions, we advise them not to attend. Yet they say they have the right to know about the truth, and publish that to the public. We have to explain once and once again that the final information is held by rescue command center, then they leave unhappily. This interferes with our work and efficiency.”*

*“Sometimes the journalists follow us everywhere we go to. Although I completely understand this is their job, they really know nothing about the specialty of our MHCI work. The priority of our work is not to interrupt the crisis clients, yet those journalists do not follow this point, always directly asking them about sensitive issues. Some residents are not willing to answer, and they still keep on asking. In fact this is harmful to the victims. I try to tell the journalists to stop every time I see, but this obviously will affect my work. I hope that media reports can consider the victims well-being more, not just think about their own benefits.”*

*“I think in recent years, after some major accidents, television news, especially the reports presented by official media, has been very objective and comprehensive. They pay attention to presenting some positive news and spirits, serving as good guidance on public opinion. There has been major improvement compared to former reports with bleeding scenes and scary numbers. However, we need to realize that the emergence of new media has widened the coverage of the audience, as well as enabling faster spreading speed and higher arbitrariness. This means the media is like a double-edged sword. If put to good use of, more people can know more accurate information and more knowledge about mental health, which helps to ameliorate public mood. Yet if not, or lacking supervision, then rumors and traumatic pictures will be everywhere. Not only may this lead to individual second impact, but also cause social unsteady factors. Therefore, the training of MHCI should also involve how to cooperate with media.”*

*“In the MHCI fieldwork after an earthquake, I was invited to the radio station of disaster area command center for an interview, which covers all residents affected by the earthquake, about 40 thousands people. To me, this was like a science-popularization interview. The host raised many targeted questions, guiding me to talk about many important and practical issues. Especially at the end, the host asked me to say some words to the victims as a psychological expert. I used this chance to stress the importance of public cohesion and recovery, the coexistence of danger and opportunity. Hope the public can find hope from desperation, seek friendship and kinship from calamity.”*

## 4.3 Hotline counselling

*“Once we arrived at the accident site, we applied to rescue command center to open two hotlines for psychological counselling. Then, every time after talking with the clients, we would leave them with the hotline number, so that they can receive help at any difficult time. Of course, people hired to answer the phone have special requirements. They need to be familiar with telephone counselling, as well as updating the newest information. That is, not only should they comfort the callers, but also help them to obtain effective information. ”*

*“Since the outbreak of the accident, these two hotlines have been preserved till now, which are specialized for the victims of this accident. A little girl lost her mother in that earthquake, and I was escorting this kid. I told her this phone number when I left, telling her that she could contact me whenever needing for help. Now, she calls me every week, telling me about her situation this week. For some people, transient on-site rescue is not enough to heal their inner trauma. What they need is long-term care, and a hotline is a very good form.”*

*“Hotlines are good. They not only help to handle crisis situations, but also strengthen later periods of psychological rehabilitation work. However, this job requires people, material, and money. If these cannot be guaranteed, this job is hard to continue for long. Therefore, this cannot be decided by MHCI workers.”*
